# Enhanced Bacterial‐Infected Wound Healing by Nitric Oxide‐Releasing Topological Supramolecular Nanocarriers with Self‐Optimized Cooperative Multi‐Point Anchoring

**DOI:** 10.1002/advs.202206959

**Published:** 2023-02-15

**Authors:** Guowei Li, Kai Lv, Qikun Cheng, Hui Xing, Wei Xue, Wu Zhang, Qianming Lin, Dong Ma

**Affiliations:** ^1^ Department of Nuclear Medicine and PET/CT‐MRI Center The First Affiliated Hospital of Jinan University Guangzhou 510630 China; ^2^ Key Laboratory of Biomaterials of Guangdong Higher Education Institutes Department of Biomedical Engineering Jinan University Guangzhou 510632 China; ^3^ The First Affiliated Hospital of Jinan University Jinan University Guangzhou 510630 China; ^4^ School of Stomatology of Jinan University Jinan University Guangzhou 510632 China; ^5^ School of Biomedical Engineering Sun Yat‐sen University, Shenzhen Campus Shenzhen 518107 China; ^6^ School of Biomedical Engineering Sun Yat‐sen University Guangzhou 510006 China; ^7^ MOE Key Laboratory of Tumor Molecular Biology Jinan University Guangzhou 510632 China

**Keywords:** antibacterial therapy, drug‐resistant bacteria, molecular motions, nitric oxide, polyrotaxane

## Abstract

Polymeric systems that provide cationic charges or biocide‐release therapeutics are used to treat the bacteria‐infected wound. However, most antibacterial polymers based on topologies with restricted molecular dynamics still do not satisfy the clinical requirements due to their limited antibacterial efficacy at safe concentrations in vivo. Here a NO‐releasing topological supramolecular nanocarrier with rotatable and slidable molecular entities is reported to provide conformational freedom to promote the interactions between the carrier and the pathogenic microbes, hence greatly improving the antibacterial performance. With improved contacting‐killing and efficient delivery of NO biocide from the molecularly dynamic cationic ligand design, the NO‐loaded topological nanocarrier achieves excellent antibacterial and anti‐biofilm effects via destroying the bacterial membrane and DNA. MRSA‐infected rat model is also brought out to demonstrate its wound‐healing effect with neglectable toxicity in vivo. Introducing flexible molecular motions into therapeutic polymeric systems is a general design to enhance the healing of a range of diseases.

## Graphical abstract

Self‐optimized cooperative multi‐point anchoring is the sliding and rotating motions of the macrocycles in the polyrotaxane structure allowing the topological supramolecular nanocarrier to optimize their molecular conformations to anchor onto the bacterial membrane, which accelerates and strengthens the interactions with the microbial, hence enhancing the antibacterial outcome.

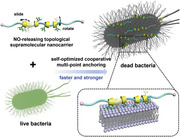

## 1 Introduction

Bacterial infections are affecting thousands of millions of people, which have been a severe global issue.^[^ ^1^ ^]^ In recent years, the abuse of antibiotics leads to the multi‐drug resistance of bacteria, which exacerbates the difficulty in combating bacterial for human‐being.^[^ ^2^ ^]^ Although many new drug therapeutics have been proposed, the low antibacterial efficiency or side effects such as cytotoxicity remain unmet medical needs.^[^ ^3^ ^]^ New biomaterial‐based therapies then are raised to address these unmet needs by providing effective delivery of drug therapeutic against bacterial with drug resistance, to avoid aggravating the evolution of drug resistance or to minimize the unconspicuous side effects.^[^ ^1^, ^4^ ^]^ Most of the antibacterial materials are designed based on two mechanisms for their antimicrobial activity: i) contacting‐killing by the materials that destroy the bacterial membranes, DNA and etc.;^[^ ^5^ ^]^ ii) releasing of biocides that are encapsulated in the materials or covalently functionalized to the materials.^[^ ^6^ ^]^ Use of antibacterial polymers, such as polycations, may be particularly appealing due to their intrinsic antibacterial activity from their cationic features, the tunability of their molecular structures and properties, and their architectures to serve as a depot for biocides.^[^ ^7^ ^]^ However, the antibacterial efficiency of polycations still does not satisfy the clinically demanding requirement at their safe concentrations otherwise unexpected irritation and inflammation may be induced at a more concentrated condition. Moreover, the eco‐ and cytotoxicities of the common biocides remain a prohibitive concern, which prevents them from continuous large‐scale use.

To promote the antibacterial outcome of the polymers either via contacting‐killing or releasing of biocides, the interactions between the polymers and the membranes of bacteria should be strengthened. First, the enhanced interactions will improve the adsorption of the cationic molecular entities onto the membrane of pathogenic microbes and then allow the effective disruption of the membrane, which leads to the death of pathogenic microbes.^[^ ^8^ ^]^ Second, in the case of releasing biocides, the strengthened interactions will promote the delivery of biocides so as to increase the bioavailability of drugs.^[^ ^9^ ^]^ In both cases, the potential toxicity and side effects can be decreased since less concentrated polymers or biocides are needed to ensure an effective antimicrobial outcome.^[^ ^10^ ^]^ In most designs, either surface charge density,^[^ ^11^ ^]^ sizes of nanoparticles,^[^ ^12^ ^]^ functional groups modification^[^ ^13^ ^]^ or variation of molecular architectures^[^ ^14^ ^]^ have been proposed to enhance the interactions between the antibacterial materials and the targeted pathogenic microbes. However, the gap between the antimicrobial performance of these polymers and medical needs is necessary to be bridged.^[^ ^15^ ^]^ Structural designs with the introduction of topologies^[^ ^16^ ^]^ that provides a controllable dynamic at the molecular level are suggested to further address the unmet need.

**Figure 1:**
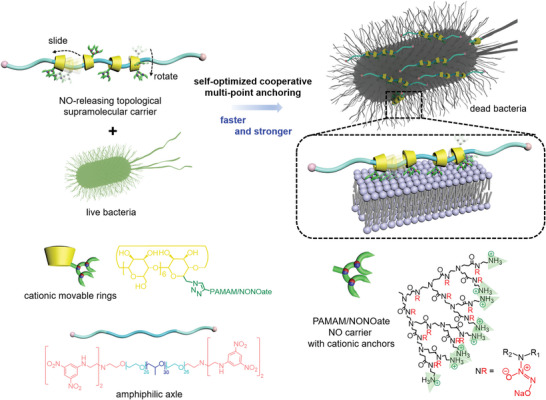
Schematic illustration of the NO‐releasing topological supramolecular nanocarrier. The sliding and rotating motions of the multiple rings modified with cationic dendrimer and NONOate in the polyrotaxane structure allow the nanocarrier to optimize their molecular conformations to anchor onto the bacterial membrane, defined as self‐optimized cooperative multiple‐point anchoring, which accelerates and strengthen the interactions between the carrier and microbial, hence enhancing the antibacterial outcome.

To this end, we designed a nitric oxide‐releasing topological supramolecular nanocarrier, with slidable and rotatable molecular entities to enhance the interactions between the nanocarrier and pathogenic microbes (**Figure** **1**). The high contacting‐killing outcome was achieved using a mechanically interlocked architecture, polyrotaxane (PR), composed of amphiphilic Pluronic polymer axles and functionalized cyclodextrin (CD) rings, to optimize the adsorption of the poly(amidoamine) (PAMAM) dendrimers with a densely cationic feature grafted on the movable rings, and the disruption of the bacterial membrane by the hydrophobic segment of the axle molecule. High releasing of biocides performance was achieved by the efficient delivery of nitric oxide (NO) released from the N‐diazeniumdiolates (NONOates) to regulate biofilm dissipation^[^ ^17^ ^]^ as a messenger molecule and to form highly reactive substances to directly destroy bacterial outer membrane,^[^ ^18^ ^]^ and metabolic enzymes,^[^ ^11a^ ^]^ and DNA, hence bypassing the barrier of multiple drug resistance^[^ ^19^ ^]^ raised by the formation of bacterial biofilm,^[^ ^20^ ^]^ weakening of drug permeability,^[^ ^20a^ ^]^ production of bacterial hydrolase,^[^ ^20b^ ^]^ change in the target of antibiotic action.^[^ ^21^ ^]^ Our key hypothesis is that the controllable dynamic at the molecular level in the designed nanocarrier would accelerate and strengthen the interactions between the microbial and the carrier so as to promote the antibacterial performance via the combination of contacting‐killing and releasing of biocides for the treatment of bacterial infection.

## 2 Results and Discussion

### 2.1 Topological Supramolecular Nanocarrier Design

We designed and successfully synthesized the topological supramolecular nanocarrier composed of several key molecular entities (Figure 1): 1) a polyrotaxane structure with movable rings providing large conformational freedom to accelerate and enhance the interactions between the carriers and microbial, allowing the grafted ligands on the movable rings to optimize their molecular conformations to anchor onto the microbial membrane, defined as self‐optimized cooperative multi‐point anchoring; 2) amphiphilic axles Pluronic polyethylene oxide‐polypropylene oxide‐polyethylene oxide (PEO‐PPO‐PEO) copolymer in which the PEO segments induce the water solubility and the hydrophobic PPO segments are able to disrupt the bacterial membrane; 3) dendritic PAMAM‐NONOates with high NO payload and releasing capability; 4) cationic primary amine end groups in the PAMAM‐NONOates dendrimers as the anchors of the movable CDs, which will insert into the negative‐charged bacterial membrane via electrostatic interactions. We selected *β*‐CD as the macrocycle component because they thread onto the middle hydrophobic PPO selectively. We hypothesized that by marrying the cationic movable rings with hydrophobic PPO segments in the mechanically interlocked structure, the disruption of the membrane caused by the hydrophobic entities could be further enhanced by the formed compact structure. At the molecular level, in the antibacterial process induced by the block copolymers, the cationic blocks are first inserted into the anionic membranes and then the hydrophobic blocks interrupt the membranes and both stages contribute to the antibacterial outcome. In the case of conventional block copolymers, the hydrophobic and cationic blocks are linked by covalent bonds. However, in the polyrotaxane structure, the cationic ring molecules can be mechanically interlocked in the hydrophobic blocks, in which the cationic rings are shuttling along the hydrophobic blocks, which affords the modified hydrophobic blocks with the capabilities to insert and interrupt into the bacterial membrane. Since the mechanical interlocked structure combines the cationic and hydrophobic features into a single block, the system is able to form a compact structure that cannot be harnessed by the conventional block copolymers.

**Figure 2:**
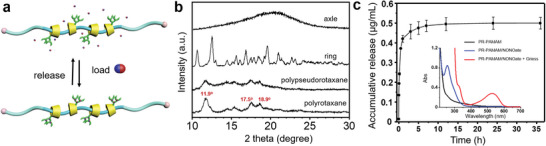
Structural characterizations of the topological supramolecular nanocarrier. a) Schematic illustration of the loading/releasing of NO molecules to/from the topological supramolecular nanocarrier. b) The PXRD profiles of the axle, ring component (*β*‐CD‐N_3_), polypseudorotaxane formed by the axle and *β*‐CD‐N_3_, and polyrotaxane (PR). c) NO‐releasing profile of PR‐PAMAM/NONOate in PBS solution at 37 °C. Inset: UV–vis spectra of PR‐PAMAM (black) and PR‐PAMAM/NONOate (blue) and PR‐PAMAM/NONOate in the sodium citrate buffer (pH 4.0) after treatment with Griess reagent (red).

The designed PR‐PAMAM was synthesized by 1) threading azide‐mono‐substituted *β*‐CD (*β*‐CD‐N_3_) onto the PEO‐PPO‐PEO copolymers to form the polypseudorotaxane; 2) an end‐capping reaction to generate the polyrotaxane; and 3) a click‐reaction to modify the third‐generation dendritic PAMAM onto the CD rings (Figure S1, Supporting Information). The binding isotherm (Figure S7, Supporting Information) showed that the azide‐mono‐substituted *β*‐CD can still thread onto the axle though the modifications on the hydroxy groups in the cyclodextrins would weaken the hydrogen bonding formation between the threaded rings.^[^ ^22^ ^]^ Powder X‐ray diffraction (PXRD) was used to index the diffraction of the formed PR structure, in which the characteristic peaks at 2*θ* = 11.9°, 17.5°, and 18.9° indicated that the typical tubular PR structure that is similar to the polypseudorotaxane before the end‐capping reaction (**Figure** **2**b).^[^ ^23^ ^]^ The nuclear magnetic resonance (NMR) spectra (Figure S8, Supporting Information) showed that ≈13 *β*‐CD‐N_3_ were mechanically interlocked onto one PEO‐PPO‐PEO axle, affording the threading ratio is ≈40% that provides the residue 60% empty space for ring's free shuttling and rotating in the PR structure.^[^ ^24^ ^]^ The cationic PAMAM dendrimer was clicked onto the mechanically interlocked *β*‐CDs through copper(I)‐catalyzed alkyne‐azide cycloaddition. The NMR profiles (Figure S9, Supporting Information) demonstrated the successful coupling and indicated that on each axle, on average 9 mechanically interlocked rings were functionalized with dendritic PAMAM successfully.

The final product PR‐PAMAM/NONOate was generated by oxidating all the secondary amines in the PR‐PAMAM under the high pressure of NO atmosphere (Figure S11, Supporting Information) while the primary amines were reserved as cationic end groups as anchors for microbial membrane‐insertion. The in‐vitro measurement of NO release of PR‐PAMAM/NONOate was conducted in the phosphate‐buffered saline (PBS) at 37 °C using the Griess reagent.^[^ ^19^, ^25^ ^]^ The strong adsorption peak at 252 and 540 nm in the ultraviolet‐visible (UV–vis) spectra indicated the presence of the NONOate structure and the released NO, respectively (Figure 2c). The total payload of NO in PR‐PAMAM/NONOate was as high as 0.495 µmol mg^−1^ (Figure S12, Supporting Information). The topological supramolecular NO‐carrier demonstrated a relatively rapid releasing rate of NO in the starting 3 h followed by a stable and slow releasing stage in the later 21 h. Both releasing stages were considered beneficial to the antibacterial performance: the burst of NO in the early stage ensures the effective concentration for damaging bacteria cells^[^ ^26^ ^]^ while the subsequent slow and sustained releasing could further inhibit the bacterial migration and multiply for a long time.

### 2.2 In‐Vitro Antibacterial Performance Enhanced by the Controllable Dynamic at the Molecular Level

**Figure 3:**
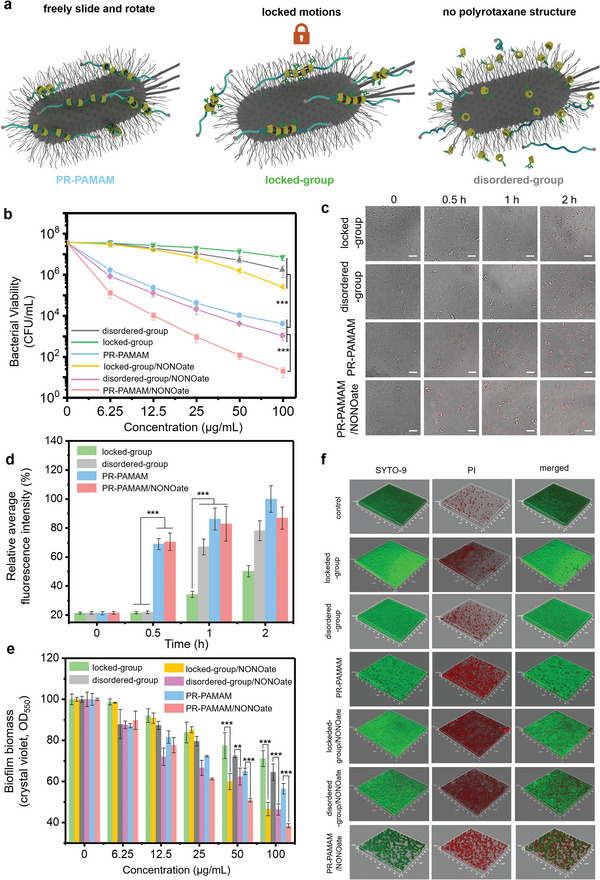
In‐vitro antibacterial performance. a) Schematic illustration of the topological supramolecular nanocarrier PR‐PAMAM/NONOate with free ring motions, the nanocarrier with crosslinked rings with limited ring motions (locked‐group) and a mixture of the molecular components in PR‐PAMAM including *β*‐CD‐PAMAM, *β*‐CD‐N_3_, DNFB‐PEO‐PPO‐PEO‐DNFB but without the polyrotaxane structure (disordered‐group), respectively. b) Viability of the bacteria treated with the different groups including the disordered group (grey), locked group (green), PR‐PAMAM (blue), disordered‐group loaded with NO (disordered‐group/NONOate, maganta), locked‐group/NONOate (yellow), locked‐group PR‐PAMAM/NONOate (pink), respectively. c) Confocal images of the bacteria with different treatments at different times. d) Relative average fluorescence intensity calculated from the confocal images of the bacteria treated with locked‐group (green), disordered‐group (grey), PR‐PAMAM (blue), and PR‐PAMAM/NONOate (pink), respectively at 0, 0.5, 1 and 2 h. e) Bacterial biofilm eradiation effects against MRSA of the disordered group (grey), PR‐PAMAM (blue), and PR‐PAMAM/NONOate (pink). f) 3D fluorescence scanning of the MRSA with different treatments. Scale bars = 20 µm.

Unlike the conventional antimicrobial polymers with structures limited by their fixed covalent bonds, the topological supramolecular nanocarrier PR‐PAMAM processes slidable and rotatable molecular entities to optimize their molecular conformations to accelerate and strengthen the interactions between the carriers and pathogenic microbes. To evaluate the antibacterial performance promoted by the introduced molecular dynamic endowed by the polyrotaxane structure, a polymer with identical molecular entities in PR‐PAMAM but crosslinked by EPI (Figure S6, Supporting Information) to restrict the rings’ molecular sliding and rotating motions, defined as the locked‐group, to restrict the rings’ molecular sliding and rotating motions was synthesized and compared with PR‐PAMAM with free motions. In addition, a mixture of the molecular components in PR‐PAMAM including *β*‐CD‐PAMAM (Figure S5, Supporting Information), *β*‐CD‐N_3_ (Figure S2, Supporting Information), dinitrofluorobenzene‐substituted‐axle (DNFB‐PEO‐PPO‐PEO‐DNFB) (Figure S4, Supporting Information) was prepared at the identical ratio in PR‐PAMAM and employed to mimic the uncombined and disordered state, which was defined as the disordered‐group. Methicillin‐resistant Staphylococcus aureus (MRSA) was treated with the obtained materials to evaluate their antibacterial performance against drug‐resistant bacteria (**Figure** **3**b). As concentrations increased, the number of MRSA survivors decreased, suggesting that the synthesized cationic polymers or mixtures in different structural states had a certain antibacterial effect. The residue 30% azide groups in the rings of polyrotaxane or cyclodextrin counterparts did not cause significant bacterial death. First, the topological supramolecular carrier PR‐PAMAM demonstrated more than 100 times higher antibacterial efficiency than the disordered group, highlighting the cooperative effect of the polymeric structure.^[^ ^27^ ^]^ Second, at the concentration of 100 µg mL^−1^, the MRSA viability of the group treated in the locked group with restricted motions is ≈1750 times higher than those treated with PR‐PAMAM with free motions. Hence, when the sliding and rotating motions were restricted by the introduced EPI crosslinker, the locked group presented much weaker antimicrobial efficiency than the PR‐PAMAM. Since the zeta‐potentials indicating the cationic charge densities in PR‐PAMAM, locked‐group, and free‐group were very close (Figure S10, Supporting Information), the promoted antibacterial performance was initially attributed to enhanced interactions by the topological structure of PR‐PAMAM with movable rings providing the conformational freedom. This was further confirmed by the investigation of adhesion to bacteria. A fluorescent dye Cy5.0 was employed to label the products (Figure S14a, Supporting Information) to observe the interactions between the antibacterial materials and bacteria at different times under a confocal laser scanning microscopy (CLSM) (Figure 3c) and the relative average fluorescence intensity was monitored (Figure 3d). They showed that compared with the bacteria treated with the disordered group or locked group, a much stronger fluorescence was found in the bacteria treated with PR‐PAMAM at each time point, suggesting the enhanced interactions^[^ ^28^ ^]^ between the topological supramolecular carrier PR‐PAMAM and the MRSA. Moreover, the topological nanocarrier PR‐PAMAM was able to enter the bacteria within 0.5 h, while the locked group and disordered group needed 1–2 h to transport into the bacteria. These results suggested that the sliding and rotating dynamic provided by the supramolecular topological design not only can enhance the interactions between the bacteria and the topological nanocarrier at thermodynamic but also accelerate the kinetic of interaction which can be beneficial to the purpose of rapid sterilization.^[^ ^29^ ^]^

### 2.3 Enhanced Antibacterial and Biofilm Dispersal Performance Promoted by the Effective Delivery of NO

Since the sliding and rotating molecular motions in PR‐PAMAM provided conformational freedom to optimize their multivalent interactions with pathogenic microbes, the topological supramolecular nanocarrier achieved an effective contact‐killing effect against drug‐resistant bacteria. On this basis, the nanocarrier was reacted with NO biocide to generate PR‐PAMAM/NONOate and the antibacterial properties were further investigated. Although NO molecules are proven to be useful in bacteria‐infected wound healing, the short half‐life of NO and their carriers prohibited their further applications.^[^ ^30^ ^]^ Hence, it is suggested that NO‐carrier is needed to deliver the NO molecules into the targets in a much more effective way compared with those existing NO‐delivery systems, such as random hyperbranched polymers, block macromolecular polymers, nanoparticles, and so on.^[^ ^31^ ^]^ We hypothesized that due to the accelerated and enhanced interactions provided by the introduced threading and rotating molecular motions, the loaded NO will be effectively delivered to bacteria so as the further improve the antibacterial effect via releasing of biocides. The locked group and disordered group were also loaded with NO to afford the locked‐group/NONOate and disordered‐group/NONOate as a comparison. As shown in Figure 3b, benefited from the released NO, the locked‐group/NONOate, disordered‐group/NONOate, and PR‐PAMAM/NONOate demonstrated improved antibacterial performance in comparison with their counterparts without loaded with NO. Compared with locked‐group/NONOate and disordered‐group/NONOate, the PR‐PAMAM/NONOate also showed the most promising antibacterial outcome. These results indicated the synergistic effect of movable ligands in the polyrotaxane structure and the efficient delivery of NO. When the concentration of PR‐PAMAM/NONOate was at 100 µg mL^−1^, the bacterial MRSA viability was as low as ≈20 CFU mL^−1^. From the fluorescence images (Figure 3c) and the relative average intensity (Figure 3d) results, the loading of NO did not weaken the interactions between the nanocarrier and bacteria. This is because the NO loading reaction only converted the secondary amine of sliding CD‐PAMAM to NONOate, leaving the cationic primary amine end groups as anchors to insert into the bacterial membrane and contribute to the effective delivery of NO. Therefore, the loss of NO due to insufficient contact with bacteria, which was a common problem of previous NO carriers,^[^ ^32^ ^]^ can be avoided and the bioavailability of NO was improved by the designed topological structure, leading to the massive mortality of MRSA.^[^ ^33^ ^]^

Bacterial biofilm is the key barrier of bacteria to resist external stimuli and improve self‐defense.^[^ ^34^ ^]^ Biofilm infections bring challenges to the clinic, including recurrent infections, chronic diseases, antibiotic resistance, etc.^[^ ^35^ ^]^ Particularly, MRSA shows resistance to various antibiotic and antibacterial agents due to the presence of biofilm. Therefore, the anti‐biofilm ability is recognized as an important aspect to determine the promise in clinical translations.^[^ ^36^ ^]^ NO is a signal regulator to regulate bacterial biofilm dissipation so it can be employed to induce the dispersal of a mature biofilm.^[^ ^37^ ^]^ Compared with other antibacterial agents, NO exhibits inconspicuous systemic side effects due to its short half‐life.^[^ ^38^ ^]^ Hence, we chose the NO as the anti‐biofilm component and investigated its performance promoted by the introduced molecular motions from the designed topology of the nanocarrier. In this study, a conventional CV staining method (Figure 3e) and 3D fluorescence scanning were performed to evaluate the anti‐biofilm activities (Figure 3f). In Figure 3e, all the evaluated materials showed a concentration‐dependent biofilm dispersal effect. When the concentration of the disordered group reached up to 100 µg mL^−1^, the biofilm biomass decreased by only 20% approximately, which was mainly attributed to the weak anti‐biofilm activity of the cationic macrocycles *β*‐CD‐PAMAM. In contrast, with the identical concentration of PR‐PAMAM, the biofilm biomass decreased by 40%, which benefited from enhanced interactions with bacteria by the self‐optimized cooperative multi‐point anchoring effect of the topological supramolecular nanocarrier itself. The anti‐biofilm performance could be further improved by the introduction of NO biocides. At the concentration of 100 µg mL^−1^, the biofilm biomass decreased by 60% and plenty of dead bacteria and thinner biofilm was found in the fluorescence images (Figure 3f), demonstrating a significantly improved anti‐biofilm effect of PR‐PAMAM/NONOate compared with the PR‐PAMAM. This enhanced performance was attributed to the effective delivery of NO in PR‐PAMAM due to strengthened interactions from the rotating and sliding molecular motions and the NO could trigger the second messenger cyclic diguanylate (di‐GMP) in the bacterial cells, activating a series of effectors that would induce the biofilm dispersal.^[^ ^39^ ^]^

### 2.4 Bacterial Membrane and DNA Damage by the Movable Cationic Ligands and Released NO

**Figure 4:**
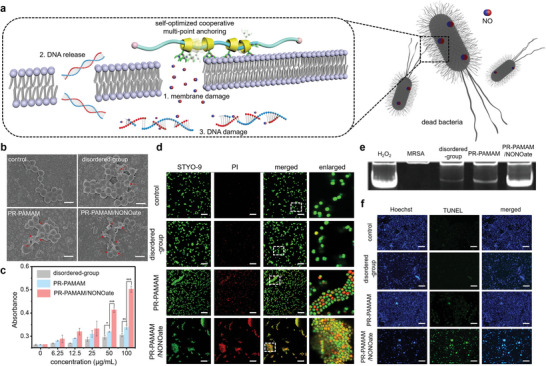
Antibacterial mechanism of the NO‐loaded topological supramolecular nanocarrier. a) Schematic illustration of the bacterial membrane damage, DNA release, and DNA damage induced by the movable cationic ligands and efficiently delivered NO into the bacteria. b) SEM images of MRSA showing the bacterial membrane deformation after different treatments. Scale bars = 1 µm. c) OD260 values of the supernatant collected from the bacterial suspensions with different treatments. d) Confocal images of MRSA with SYTO‐9/PI dual fluorescent staining recorded after different treatments. Scale bars = 20 µm. The OD260 value indicated the damage degree of MRSA. e) PAGE results of the DNA collected from the bacterial suspensions with different treatments. f) TUNEL assay indicates the DNA damage degree of MRSA by different treatments. The damaged DNA can be stained in green color in the TUNEL assay, the intensity of green fluorescence represents the DNA damage degree. Scale bars = 50 µm.

Mechanistic studies were conducted to further understand the antibacterial process of the NO‐releasing topological supramolecular nanocarrier. Since the cell membrane is the first and foremost protective barrier of the pathogenic microbes, we considered that the bacterial membrane would be first destroyed by the insertion of the cationic and amphiphilic nanocarrier, and the released NO biocide, and then they released NO residue would play its antibacterial roles to destroy the bioactive contents inside the bacterial cells (**Figure** **4**a). A scanning electron microscope (SEM) was used to directly observe the damage degree of the bacterial membrane (Figure 4b). Compared with the regular and smooth membrane of normal bacteria, bacterial membrane after different treatments crumpled and turned rough to some extent (highlighted by the red arrows). Significantly, after being loaded with NO, PR‐PAMAM/NONOate demonstrated an extensive membrane breakage capability, which would lead to the release of cellular contents such as DNA and RNA. Furthermore, OD260 of the supernatant of bacterial solution after different treatments was measured (Figure 4c) to verify the release of cellular contents due to the destroyed bacterial membrane. Compared with the disordered group, the nanocarrier itself, PR‐PAMAM, could cause more serious leakage of cellular contents because the PR‐PAMAM is able to strongly absorb bacteria through Columbian force between the movable cationic rings and anionic membranes, therefore, increasing the membrane permeability and preventing the normal physiological activities to kill the bacteria effectively.^[^ ^40^ ^]^ Moreover, the released NO from the PR‐PAMAM/NONOate induced the most significant effusion and membrane damage due to the oxidation of proteins and metabolic enzymes caused by the reactions triggered by NO.^[^ ^26^ ^]^ The membrane integrity of MRSA treated by different groups was determined by the SYTO‐9/PI double fluorescence staining (Figure 4d), in which the SYTO‐9 could penetrate both intact and damaged membranes to stain DNA while PI could only penetrate the damaged membrane.^[^ ^41^ ^]^ Without the polyrotaxane structure, the disordered groups had a slight impact on the integrity of the MRSA membrane while the PR‐PAMAM was able to effectively induce the destruction of the bacterial membrane due to the self‐optimized cooperative multi‐point anchoring effect. Moreover, the treatment of PR‐PAMAM/NONOate further promoted the damage degree of the membrane. A partial magnification of the area in the fluorescence image clearly showed that almost no intact membrane could be found. These results were consistent with those from the SEM images and the measurement of released cellular contents. Taken together, the topological supramolecular nanocarrier PR‐PAMAM/NONOate was proven to be capable of damaging bacterial membranes so as to achieve outstanding antibacterial performance owing to the synergetic effect of movable cationic rings and the releasing of NO.

Since the nanocarrier would induce bacterial membrane damage that would promote NO to penetrate the cell, we also considered that after the delivered NO may cause bacterial death through the nitrosation reaction with DNA.^[^ ^42^ ^]^ In order to verify the damage to DNA, the genomic DNA of bacteria after different treatments were collected, and a polyacrylamide gel electrophoresis (PAGE) experiment was performed (Figure 4e). H_2_O_2_ treatment was used as a positive control and the non‐treatment MSRA group was set as a negative group. Compared with the disordered group, more damaged bacterial DNA was found in the group treated by the PR‐PAMAM nanocarrier. More importantly, PR‐PAMAM/NONOates caused the most significant swearing on DNA bands indicating the serious damage of MRSA's DNA, in which the outcome was almost as high as H_2_O_2_. These results were also consistent with those in the terminal deoxynucleotidyl transferase dUTP nick end labeling (TUNEL) assay (Figure 4f). Hence, besides the membrane damage, the NO‐releasing topological supramolecular nanocarrier is capable of killing the bacteria via inducing the DNA damage promoted by the synergetic effect of movable cationic rings and releasing of NO.

### 2.5 In‐Vivo Wound Healing

**Figure 5:**
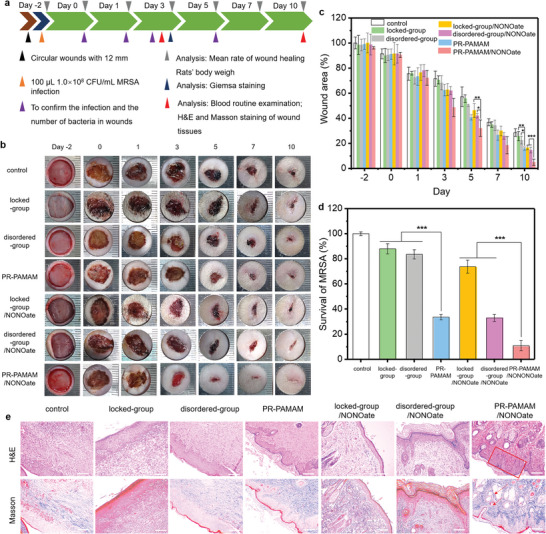
In vivo bacteria‐infected therapy. a) Schematic diagram of infected wound formation and the subsequent treatments. b) Digital images showing changes in rats’ state and wound area on different days after undergoing different treatments. c) Comparison of rats’ wound areas on different days after undergoing different treatments. d) Comparison of survival ratio of MRSA with different treatments at Day 5. e) Histological images with H&E and Masson staining of wound tissue collected on Day 10. Scale bars = 50 µm.

**Figure 6:**
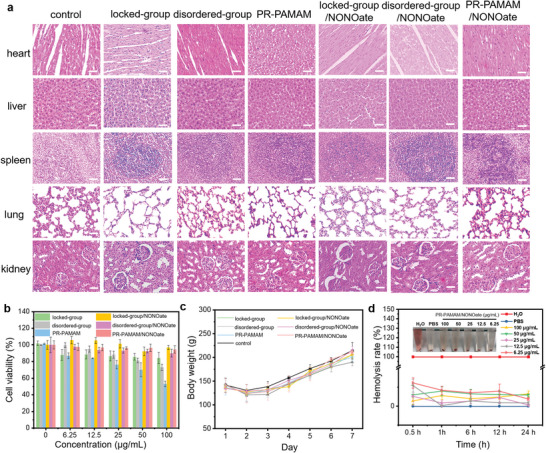
Biocompatibility of the topological supramolecular nanocarrier PR‐PAMAM/NONOate. a) H&E staining images of the heart, liver, spleen, lung, and kidney of rats after 10‐day treatments. Scale bar = 50 µm. b) The cell viability of L929 cells with different treatments at different concentrations. c) Changes in rats’ body weight of each group on different days. d) Hemolysis evaluation of erythrocytes upon the incubation with PR‐PAMAM/NONOate (6.25, 12.5, 25, 50, and 100 µg mL^−1^) for 0.5–24 h. H_2_O and PBS were used as a positive and negative control.

To investigate whether the molecularly well‐engineered NO‐loaded topological supramolecular nanocarrier could provide bacteria‐infected wound healing in vivo, studies in the rat model with MRSA‐infected wounds were conducted. (**Figure** **5**a). First, MRSA was used to induce the wound to a diameter of 12 mm and after 2 days (Day 0), a large area of yellow pus was found on the created wounds and collected for bacterial quantitation (Figure 5b). The wound area (Figure 5b,c), survival number of MRSA (Figure 5d), and body weights (**Figure** **6**c) were recorded in the 10‐day treatment with locked‐group, disordered‐group, PR‐PAMAM, locked‐group/NONOate, disordered‐group/NONOate, and PR‐PAMAM/NONOate, respectively at the identical concentration (50 µg mL^−1^). On Day 10, blood and pathological section were collected to evaluate the antibacterial and wound healing outcome.

In the wound area and bacterial number analysis, all the experimental groups showed some wound‐healing effect in vivo, which was attributed to the cationic feature of the ring components CD‐PAMAM. Compared with the locked group and disordered group, the number of bacteria treated with PR‐PAMAM for 3 days was decreased (Figure S15a, Supporting Information) and the reduction of wound area was more pronounced, highlighting the improvement via contact‐killing from the self‐optimized multi‐point anchoring endowed by the designed polyrotaxane structure. Since the decrease of bacteria number is considered beneficial for wound healing in vivo,^[^ ^43^ ^]^ the topological nanocarrier with the enhanced antibacterial activity had advantages in promoting wound healing. After being loaded with NO, the locked‐group/NONOate, disordered‐group/NONOate, and PR‐PAMAM/NONOate showed improved outcomes in wound‐healing (Figure 5b,c) and reducing the bacteria (Figure 5d) in the wound. Remarkably, the reduction of wound area and survival number of MRSA were most significant in the group treated with PR‐PAMAM/NONOate. On Day 5, the bacteria number was decreased by 90% and the wound‐healing rate was increased up to 60%. On Day 10, the NO‐loaded nanocarrier‐treated wounds healed up and no obvious scar was found. These anti‐infection results revealed that both the polyrotaxane structure with movable rings and the loaded NO contributed to the enhanced anti‐infection effect of the designed polyrotaxane.

To further evaluate the promoted wound‐healing effect of the NO‐loaded topological nanocarrier, H&E, Masson, and Giemsa staining were applied on the histological slides, respectively (Figure 5e). Numerous neutrophils, serious tissue edema, and pathogenic microorganism were found in the H&E staining images of each group on Day 3, which suggested the serious infected situation at this stage (Figure S15b, Supporting Information). Notably, in the wound treated by the PR‐PAMAM/NONOate, fibroblasts in the dermis and subcutaneous tissue proliferated and differentiated and a large number of erythrocytes existed, which were important indicators for the wound‐healing process.^[^ ^44^ ^]^ Moreover, Masson staining analysis revealed the regeneration of dermal tissue and collagen in the wound treated by the PR‐PAMAM/NONOate (Figure S15b, Supporting Information), which was attributed to the efficient delivery of NO by the designed topological supramolecular nanocarrier. On Day 10, intact dermis with abundant collagen (highlighted in the red rectangle in Figure 5e) was found in the wound treated by the NO‐loaded carrier and hair follicle tissue (pointed by the red arrow in Figure 5e) was successfully regenerated, suggesting the functional recovery of the skin.

### 2.6 Biocompatibility

Cytotoxicity assay was performed to evaluate the biocompatibility of the designed materials (Figure 6b). Compared with the disordered group and locked group, the nanocarrier PR‐PAMAM reduced the activities of L929 cells due to the enhanced nonspecific ion adsorption effect between the cationic polymers and cells caused by the polyrotaxane structure. However, after being loaded with NO, PR‐PAMAM/NONOate demonstrated improved biocompatibility and the cell retained a viability of 95% after the treatment of PR‐PAMAM/NONOate at the therapeutical concentrations. A similar trend was also found in the cells treated with locked‐group/NONOate and disordered‐group/NONOate. We attributed the reduction of cytotoxicity to the conversion of the cationic secondary amine group in PR‐PAMAM to NONOate and the promotion of cell proliferation caused by the released NO.^[^ ^45^ ^]^ Therefore, the special physiological properties of NO endowed PR‐PAMAM/NONOate with excellent cytocompatibility.

Furthermore, histological analysis was performed on slides of the main organs of the rats (heart, liver, spleen, lung, and kidney) stained with H&E, Masson, and Giemsa, respectively, to evaluate the toxicity in vivo (Figure 6a). After a 10‐day treatment, rats in therapeutic groups demonstrated no exception compared with the blank group. Moreover, the blood indexes of rats showed no significant change as well. The body weights of rats were recorded during treatments (Figure 6c). Although the body weights decreased at the initial stage of the trauma, they kept a relatively stable level similar to the trend of blank control during the whole 10‐day treatment. The in‐vivo safety was further examined by the hemolysis assay of PR‐PAMAM interacting with erythrocytes (Figure 6d). The hemolysis rates were less than 5% in each period even though the concentration of PR‐PAMAM/NONOate was as high as 100 µg mL^−1^, demonstrating the good blood compatibility of the NO‐loaded nanocarrier. These in‐vivo studies demonstrated the neglectable toxicity of the designed topological supramolecular nanocarrier.

## 3 Conclusion

To conclude, we designed and synthesized a NO‐releasing topological supramolecular nanocarrier with rotating and sliding molecular motions for antibacterial therapy. The endowed molecular motions allowed the movable ligands in the nanocarrier to optimize their molecular conformations to anchor onto the bacteria membrane, accelerating and enhancing interactions between the pathogen and the nanocarrier. The promoted interactions by the introduction of molecular dynamics largely improved the antibacterial performance of the designed cationic polymer via contacting‐killing. On the basis of a structurally well‐engineered topological nanocarrier with flexible molecular motions, a NO‐donor was constructed and excellent antibacterial and antibiofilm performance were achieved due to the synergistic effect of movable cationic rings and efficient delivery of NO. The in‐vitro analysis revealed that the cationic polyrotaxane and released NO were able to destroy the bacterial membrane and the delivered NO residue could induce the damage of DNA, leading to the mass death of MRSA. Moreover, the in‐vivo model proved effective antibacterial and wound‐healing effects for MRSA‐infected wounds. Taken together, the design of NO‐releasing topological supramolecular nanocarrier for the treatment of antibacterial therapy described here may provide a promising strategy to accelerate the clinical transformation of antibacterial polymers.

## 4 Experimental Section

### Materials Preparation

*β*‐cyclodextrin (*β*‐CD), sodium ascorbate, PEO‐PPO‐PEO triblock copolymer (poloxamer L64), paratoluensulfonyl chloride, N, N'‐carbonyldiiazole, Tri‐(2‐aminoethyl) amine, triethylamine, 2,4‐dinitrofluorobenzene (DNFB), copper sulfate pentahydrate, and sodium methylate were purchased from Shanghai Aladdin Biochemical Technology Co., Ltd. All reagents were used directly without further purification.

MRSA was obtained from the center of bacterial identification of the dermatology department, the First Affiliated Hospital of Jinan University. Tryptic soy broth (TSB) and Luria‐Bertani (LB) agar were purchased from Guangdong Huankai Biological Technology Co., Ltd and stored in a 4°C refrigerator. A cell counting kit‐8 (CCK‐8) was purchased from Tongren Co., Ltd. Live/Dead BacLight bacterial viability kit (L7012) was bought from Thermo Fisher Technology Co., Ltd. Dulbecco's modified eagle medium, fetal bovine serum (FBS), and tryptase were purchased from Gibco Life Technologies. Total Nitric Oxide Assay Kit was purchased from Shanghai Biyuntian Biotechnology Co., Ltd.

### Characterization

For structural characterization, the ^1^H spectra of the obtained polymers dissolved in deuterated reagent were recorded on a Bruker 300 NMR spectrometer at 300 MHz and analyzed using MestReNova software. An appropriate amount of materials were used for FTIR measurement using KBr in transmission mode on a VERTEX 70 spectrometer (Bruker Dalton, German). For mass spectrographic analysis, moderate *β*‐CD‐N_3_ was tested in the Na^+^ mode with the ion temperature of 125 °C, a deionizing temperature of 250 °C, a desolvation gas flow of 400 L h^−1^, cone gas flow of 50 L h^−1^, the capillary voltage of 2.5 kV, cone voltage of 50 V, and the mass spectrum range was 500–1500 m z^−1^. Moderate PR‐PAMA and PR‐PAMAM/NONOate were respectively dissolved in ultrapure water at the concentration of 1 mg mL^−1^ for the UV spectra measurement at room temperature, and their spectra in the range of 200–600 nm were recorded using a UV–vis spectrophotometer. For the isothermal titration calorimeter (ITC) measurement, *β*‐CD‐N_3_ and PEO‐PPO‐PEO tetra(amine) were dissolved in ultrapure water respectively, of which the concentration ratio was 20:1. Samples, solvent, and volumetric solution were vacuumed using a vacuum pump to remove the bubbles inside. After adding *β*‐CD‐N_3_ into the injection of ITC, PEO‐PPO‐PEO tetra(amine) was added to the sample cell and water was added to the reference cell. Parameters were set as 50 × 2 µL, every drop interval as 200 s, and rotation speed as 100 rpm, 25 °C. Zeta potentials of different nanomaterials including PR‐PAMAM, locked‐group, and disordered‐group were measured on a Zetasizer Nano ZS (Malvern) apparatus equipped with a zeta potential analyzer software.

### Synthesis of PR

The synthesis of PR was divided into two steps. First, multiple *β*‐CD‐N_3_ rings were threaded on the aminated PEO‐PPO‐PEO triblock copolymer to form the mechanically interlocked polymers which were named poly(pseudo)rotaxanes (PPR).^[^ ^24a^ ^]^ Then, PR was prepared by end‐capping PPR using stopper molecules. First of all, the aminated PEO‐PPO‐PEO (0.4 g) was dissolved in 266 mL of *β*‐CD‐N_3_ aqueous solution (0.03 g mL^−1^) by adding NaHCO_3_ (0.6 g) to regulate the pH. After the ultrasound treatment for 20 min and the following reaction overnight, the PPR was successfully prepared by lyophilization. In the second step, DNFB was employed as the end‐capping reagents according to a report.^[^ ^46^ ^]^ In Brief, the obtained PPR was dissolved in 25 mL of anhydrous DMF, and then DNFB (0.39 g) was added. After the reaction in the N_2_ atmosphere overnight, a further reaction at 80 °C for 2 h was carried out. The mixture was precipitated and washed with ether repeatedly to remove the unreacted DNFB. After the solution was dried and dissolved in DMSO, it was precipitated and washed with methanol to remove by‐products, and then it was redissolved in DMSO. Eventually, the solution was precipitated and washed with pure water several times to obtain PR as a yellow powder (yield, 89%).

### Synthesis of Cationic PR‐PAMAM

According to our reported method, PAMAM‐G3 was successfully prepared.^[^ ^41^ ^]^ PAMAM‐G3 was grafted onto PR by a click reaction. Briefly, PR (1 g) was dissolved in 80 mL of DMSO followed by adding 10 mL of PAMAM‐G3 aqueous solution (0.206 mmol mL^−1^) and copper sulfate pentahydrate (40 mg). After stirring for 30 min, 10 mL of sodium ascorbate aqueous solution (20 mg mL^−1^) was added and the reaction temperature was elevated to 70 °C. After 72 h, the solution was dialyzed against water in a dialysis bag (MW 5000 Da) for 3 days. PR‐PAMAM was obtained after lyophilization (yield, 49%).

### Synthesis of NONOates

At room temperature, PR‐PAMAM, locked‐group, and disordered‐group (200 mg) were dissolved in 15 mL of the mixed solvent of methanol and pure water (v/v 1:1 mixture) with full stirring. Sufficient sodium methoxide whose molar weight was equal to that of the secondary amine groups on PAMAM was added and the solution was transferred to a miniature high‐pressure reactor kettle. After examining the tightness, it was purged with N_2_ and maintained at 20 psi for 30 min. Then, the rector kettle was inflated with NO gas and maintained at 80 psi for 3 days. After the reaction finished, NO gas was thoroughly removed by flushing N_2_ (50 psi) before the resulting solution was taken out. The solution was poured into acetone to precipitate, which was washed with ether 3 times. The resulting precipitate was dried in a vacuum at room temperature to give PR‐PAMAM/NONOate (yield, 87%) stored at −20 °C for further use.

### Determination of NO Loading Content

In this study, a Griess reagent kit was used to measure the NO payload of PR‐PAMAM/NONOate.^[^ ^47^ ^]^ In brief, PR‐PAMAM/NONOate (1 mg) was dissolved in 5 mL of citrate buffer solution (pH 4.0) and incubated in a 37 °C shaker for the complete release of NO. After 4 h, an appropriate amount of the mixture was taken and mixed with Griess reagent. After incubating in dark for 15 min, the OD540 was measured to calculate the NO loading content in PR‐PAMAM/NONOate according to the established standard curve.

### In‐Vitro NO Release

PR‐PAMAM/NONOate (10 mg) was dissolved in 5 mL of PBS (pH 7.4) and enclosed in a dialysis bag (500 Da), which was immersed in 45 mL of PBS and incubated in a 37 °C shaker. At interval time points (1 min, 5 min, 10 min, 0.5 h, 1 h, 3 h, 5 h, 7 h, 12 h, 16 h, 24 h, and 36 h), the release solution was shaken fully and 5 mL of it was taken out and replaced by 5 mL of fresh PBS. The aliquot was mixed with Griess reagent and incubated in dark for 15 min. Then the OD540 was measured and the percentage of the released NO was calculated. Each point was performed 3 times, and the result was shown as an average and error value.

### Bacteria Storage and Preparation

The clean bench and materials were sterilized by a UV lamp. All things on the clean bench were disinfected with 75% alcohol. Before bacterial culture, the culture medium, culture dish, and other vessels were sterilized. MRSA strains were obtained from the first affiliated hospital of Jinan University and cultured according to standards. Briefly, a single colony of MRSA on the agar plate containing methicillin sodium was taken using an inoculating loop, and it was added to the TSB medium containing methicillin sodium (5 µg mL^−1^). After the incubation at 37 °C incubation for 24 h, the culture medium was removed by centrifugation, and then the freezing medium was mixed with bacteria in a frozen pipe, which was stored at −80 °C for further use. The frozen bacterial solution was melted in a 37 °C bath and transferred into a TSB medium containing methicillin sodium by an inoculating loop. After culturing at 37 °C overnight, the bacterial suspension was taken by an inoculating loop and added to fresh TSB containing methicillin sodium. The bacteria were cultured for 4 h until the logarithmic phase for use.

### Antibacterial Assays

The mixture solution of PEO‐PPO‐PEO‐DNFB, *β*‐CD‐PAMAM, and *β*‐CD‐N_3_ was set as the free group. EPI‐PR‐PAMAM with *β*‐CD cross‐linked to limit the molecule mobility of *β*‐CD on the PEO‐PPO‐PEO was also set as the locked group. MRSA at the logarithmic phase was centrifuged to remove the culture medium and it was resuspended in PBS. The bacterial concentration was adjusted to 1 × 10^8^ CFU mL^−1^ and seeded in 96 plates. Bacteria were treated with PR‐PAMAM/NONOate and PR‐PAMAM (6.25, 12.5, 25, 50, and 100 µg mL^−1^). After co‐culturing at a 37 °C shaker for 4 h, the co‐culture solution was centrifuged and washed before the gradient dilution. Then the diluted solution was spread on sterile LB agar and cultured at a 37 °C incubator overnight. Finally, the number of colonies was counted to evaluate the antibacterial performance of PR‐PAMAM/NONOate.

### Adhesion to Bacteria

The fluorescent dye Cy5.0 was used to label the free‐group, locked‐group, PR‐PAMAM, and PR‐PAMAM/NONOate. A laser scanning confocal microscope (CLSM) was used to observe the interaction between materials in different groups and bacteria at different times. In brief, PR‐PAMAM/NONOate, PR‐PAMAM, free‐group, and locked‐group (10 mg) were dissolved in 5 mL of pure water, and then 40 µL of the Cy5.0 DMSO solution (5 mg mL^−1^) was added. After stirring in the dark overnight, the solution was dialyzed and freeze‐dried. A fluorescence spectrophotometer was used to record the fluorescence spectra of the cy5.0 labeled free‐group, locked‐group, PR‐PAMAM, and PR‐PAMAM/NONOate. Then, the Cy5.0 labeled materials were re‐suspended in PBS at the concentration of 50 µg mL^−1^, and mixed with MRSA solution (1 × 10^4^ CFU mL^−1^) for a certain time (0 min, 30 min, 1 h, and 2 h). After that, MRSA was collected by centrifuging, and it was washed with PBS to remove materials. Eventually, MRSA was suspended in PBS and observed using a CLSM. Cy5.0 showed red fluorescence under the 650/670 nm excitation.

### Antibacterial Biofilm Assays

MRSA at the logarithmic phase was seeded into 24 plates (100 µL well^−1^). Then, 1 mL of culture medium was added for the incubation lasting 48 h. The culture medium was refreshed every 12 h to form dense and mature biofilm.

### Antibiofilm Activity of PR‐PAMAM/NONOate

To study the biofilm dispersal effect of different materials with different structures, materials were added into cells where biofilm formed. Briefly, preformed biofilm was subjected to different treatment groups: PBS, free‐group, PR‐PAMAM, and PR‐PAMAM/NONOate. Concentrations of materials were 6.25, 12.5, 25, 50, and 100 µg mL^−1^. After treatments for 12 h in a 37 °C incubator, each well was washed with PBS to remove materials and the floating bacteria, followed by adding 500 µL of methanol for fixation. Then, 1% of crystal violet (CV) dye was added to stain the biofilm. After staining in dark for 30 min, samples were washed with sterile water, and then cells were dried at room temperature before photographing. After that, absolute ethyl alcohol was added to dissolve the crystal violet dye, and OD540 was measured.

### 4.1 Fluorescence Scanning

To reveal the anti‐biofilm effect of different materials, biofilm was stained with fluorescent dyes SYTO‐9/PI after different treatments for a certain time in the dark, and then the redundant dyes were washed off with water. Finally, the stained biofilm was observed by a laser scanning confocal microscope (CLSM) and was photographed in 3D mode.

### Live/Dead Fluorescence Staining

MRSA at the logarithmic phase was centrifuged to remove the culture medium and re‐suspended in PBS, whose concentration was adjusted to 1 × 104 CFU mL^−1^. Four groups were set: blank control group, free‐group, PR‐PAMAM group, and PR‐PAMAM/NONOate group. After the co‐incubation at a 37 °C shaker for 4 h, materials were removed by centrifugation. Then live/dead fluorescent dye (SYTO‐9/PI) was added and incubated in dark. After 15 min, the excess dye was removed by washing with PBS. A laser scanning confocal microscope was used to observe photographing. Note that, SYTO‐9 glows green fluorescence under 539/570‐620 nm excitation, while PI glows red under 470/490‐540 nm excitation.

### Scanning Electron Microscope Observation

SEM was used to observe the bacterial morphology after different treatments. In brief, MRSA was cultured to the logarithmic phase and suspended in PBS after removing the culture medium by centrifugation. Then, materials in the free group, PR‐PAMAM group, and PR‐PAMAM/NONOate group were added and incubated at a 37 °C shaker. The PBS‐treated group was set as a control. After that, materials were removed by centrifugation, and bacteria were collected, followed by the fixation using 2% pentanediol at 4 °C for 3 h. Ethanol at gradient concentrations (30, 50, 60, 70, 80, and 100%) was used for dehydration for 10 min respectively. Finally, the treated bacteria were set on a San EM holder and coated and sprayed by Au before observation.

### UV–Vis Measurement of Inclusion Oozed from Bacterial Cells

After different treatments, OD260 of the inclusion oozed from bacterial cells was measured to evaluate the bacterial cell membrane integrity. Briefly, MRSA at the logarithmic phase was re‐suspended in PBS, followed by adding materials in the free group, PR‐PAMAM group, and PR‐PAMAM/NONOate group. PBS treatments were set as a control. After incubation at a 37 °C shaker, bacteria suspension was filtered by a 0.22 µm membrane to remove materials. Finally, OD260 of the bacterial supernatant was measured using a UV spectrophotometer.

### Bacterial DNA Damage Evaluation by PAGE Experiment

Bacteria were suspended in PBS at the concentration of 10^6^ CFU mL^−1^ and different materials were added. After incubation, bacteria were collected by centrifugation and re‐suspended in PBS. According to the instructions, bacterial Genomic DNA Kits (GenElute) were used to extract the DNA of the treated bacteria. The collected DNA was separated by the PAGE experiment, of which the working voltage was 150 V for 3 h. Bacteria treated with H_2_O_2_ were set as control.

### Bacterial DNA Damage Evaluation by Transferase‐Mediated Nick end Labeling (TUNEL) Assay

Bacteria were suspended in PBS at the concentration of 10^6^ CFU mL^−1^ before different treatments. After being treated with different materials, bacteria were collected by centrifugation and stained by TUNEL using a staining kit. Finally, the TUNNEL staining bacteria were observed using a fluorescence microscope.

### Modeling of MRSA‐Infected Wounds

Male SD rats (Average body weight was 200 g) were purchased from Southern Medical University Center for Animal Experiments (Guangzhou, China). All the rats were specifically pathogen‐free and raised in the Experimental Animal Center of Jinan University. The Institutional Administration Panel for Laboratory Animal Care confirmed all animal experiments (Medical Ethics Committee of Jinan University). Male SD rats were divided into four groups with five rats in each group. Before experiments, they were raised in a sterile environment with natural light‐dark cycles for 7 days. After intraperitoneal injection of 10% chloral hydrate, squared operative regions were created by shaving on the back of rats with the spine as the central axis. The depilatory paste was used for further depilation. After sterilizing, two 12‐mm wounds were created in each rat by dermis excision. MRSA solution (150 µL, 10^7^ CFU mL^−1^) was added to each wound. After 2 days, agar plates were used to confirm the successful modeling of MRSA‐infected wounds.

### Assessments for Antibacterial and Healing Effects

Rats were divided into four groups, and rats in each group were treated with PBS, free‐group, PR‐PAMAM, and PR‐PAMAM/NONOate respectively. All materials were dissolved in PBS at the concentration of 50 µg mL^−1^ before being sprayed on wounds every day. On days 1, 3, 5, and 7, a certain volume of leaching solution of each wound was taken and diluted 100 times for the CFU quantification analysis using LB agar plates, in order to record the bacterial amount in wounds to compare the antibacterial effect of different materials. Meanwhile, on days 1, 3, 5, and 7, the digital camera was used to photograph wounds. An aperture measuring scale was used to measure wounds.

### Histological Image Analysis

On day 3, two rats in each group were executed. Wound tissue was taken and mixed in 4% paraformaldehyde, and then paraffin embedding, H&E staining, Masson staining, and Giemsa staining were performed. Finally, an optical microscope (Motic‐BA310) was used to observe the histologic images. On day 10 when the treatment was finished, the same management except Giemsa staining on day 3 was done to the rest rats.

### In‐Vitro Cytotoxicity

Mouse fibroblast cells L929 were used to evaluate the cytotoxicity of PR‐PAMAM/NONOate. In brief, L929 cells at the logarithmic phase were digested and collected to resuspend in DEME complete culture containing 10% FBS and 1% penicillin‐streptomycin. Cells were seeded in a 96‐cells plate with a cell density of 1 × 10^4^ each cell and were cultured in a cell incubator with a 5% CO_2_ atmosphere at 37 °C overnight. After that, the culture medium was replaced with a fresh medium containing different concentrations of the free group, PR‐PAMAM, and PR‐PAMAM/NONOate. After 24 h, cell activities were measured using a CCK‐8 kit according to the operation guide. Fresh medium was used as a control, and there were five duplicates in each group. For the evaluation of treatment safety, during the treatment process, a precision balance was used to record the body weight of each rat on days −2, 0, 1, 3, 5, 7, and 10.

### Hemolysis Assay

PR‐PAMAM/NONOate (6.25, 12.5, 25, 50, and 100 µg mL^−1^) was added to 50 µL of erythrocyte suspension (16% in PBS, v/v). The negative and positive controls were PBS and distilled water, respectively. The mixtures were cultured at room temperature for a given period (0.5, 1, 6, 12, and 24 h). Then the supernatant was collected and added to a 96‐well plate. The OD540 of hemoglobin in the supernatant was measured. The formula for calculating hemolysis rate: Hemolysis(%) = (A − C)/(B − C) × 100%. A, B, and C represent the OD540 of the supernatant in the PR‐PAMAM/NONOate group, positive control, and negative control, respectively.

### Evaluation of In‐Vivo Biocompatibility

For the evaluation of treatment safety, during the treatment process, a precision balance was used to record the body weight of each rat on days −2, 0, 1, 3, 5, 7, and 10. Rats in all groups underwent euthanasia after treatments. Organs including the heart, liver, spleen, lung, and kidney were harvested and washed with PBS to remove blood, and then they were soaked in 10% paraformaldehyde. Paraffin embedding, Masson staining, and Giemsa staining were performed. Finally, an optical microscope (Motic‐BA310) was used to observe the histologic images.

### Statistical Analysis

Each experiment contained at least three parallel samples and presented as mean ± standard deviation. Differences between experimental groups were analyzed using GraphPad software. One‐way ANOVA test and Tukey's post hoc analysis. The levels of significant difference were marked as follows: *p* < 0.05 (*), *p* < 0.01(**), and *p* < 0.001(***).

## Conflict of Interest

The authors declare no conflict of interest.

## Author Contributions

G.L., Q.L., and D.M. designed the work. G.L. and Q.L. wrote the manuscript. G.L. performed the experiments and collected the data. K.L., Q.C., and H.X. helped with some measurements. G.L., W.X., Q.L., W.Z., and D.M. provided research funding. All authors have given approval for the final version of the manuscript.

## Supporting information

Supporting Information

## Data Availability

The data that support the findings of this study are available from the corresponding author upon reasonable request.
