# Supplementary material for: Enhanced Bacterial‐Infected Wound Healing by Nitric Oxide‐Releasing Topological Supramolecular Nanocarriers with Self‐Optimized Cooperative Multi‐Point Anchoring
Source: Adv Sci (Weinh). 2023 Feb 15;10(11):2206959. doi: 10.1002/advs.202206959 (PMC10104656; doi:10.1002/advs.202206959)
Supplement: Supplementary file 1 — Supporting Information [file ADVS-10-2206959-s001.pdf]

ADVANCED SCIENCE
Open Access

Supporting Information

for *Adv. Sci.*, DOI 10.1002/advs.202206959

Enhanced Bacterial-Infected Wound Healing by Nitric Oxide-Releasing Topological Supramolecular Nanocarriers with Self-Optimized Cooperative Multi-Point Anchoring

Guowei Li, Kai Lv, Qikun Cheng, Hui Xing, Wei Xue, Wu Zhang, Qianming Lin* and Dong Ma**

ADVANCED SCIENCE
Open Access

Supporting Information

for *Adv. Sci.*, DOI 10.1002/advs.202206959

Enhanced Bacterial-Infected Wound Healing by Nitric Oxide-Releasing Topological Supramolecular Nanocarriers with Self-Optimized Cooperative Multi-Point Anchoring

Guowei Li, Kai Lv, Qikun Cheng, Hui Xing, Wei Xue, Wu Zhang, Qianming Lin* and Dong Ma**

Enhanced bacterial-infected wound healing by nitric oxide-releasing topological supramolecular nanocarriers with self-optimized cooperative multi-point anchoring

Guowei Li [a,b], Kai Lv [b], Qikun Cheng [b], Hui Xing [b] , Wei Xue [b], Wu Zhang [c, d, *], Qianming Lin [e, f, *], Dong Ma [b, g, *]

[a] Department of Nuclear Medicine and PET/CT-MRI Center, The First Affiliated Hospital of Jinan University, Guangzhou, 510630, China

[b] Key Laboratory of Biomaterials of Guangdong Higher Education Institutes, Department of Biomedical Engineering, Jinan University, Guangzhou 510632, China

[c] The First Affiliated Hospital of Jinan University, Jinan University, Guangzhou, 510630, China

[d] School of Stomatology of Jinan University, Jinan University, Guangzhou, 510632, China

[e] School of Biomedical Engineering, Sun Yat-Sen University, Shenzhen Campus, Shenzhen, 518107, China

[f] School of Biomedical Engineering, Sun Yat-sen University, Guangzhou, 510006, China

[g] MOE Key Laboratory of Tumor Molecular Biology, Jinan University, Guangzhou, 510632, China

* Corresponding authors.

E-mail addresses: tmadong@jnu.edu.cn (Dong Ma), linqm5@mail.sysu.edu.cn (Qianming Lin), tzhangwu@jnu.edu.cn (Wu Zhang).

This PDF file includes:

Experimental Section

Results and Discussions

Supplementary Figure 1 to Figure 15

Supplementary Table 1

Supplementary References

1. Experimental Section

1.1 Synthesis of aminated PEO-PPO-PEO

PEO-PPO-PEO tetra(amine) was synthesized following the reported method with some modifications[1]. In detail, PEO-PPO-PEO triblock copolymer (Mn = 2,930, 1.493 g, 0.51 mmol) was heated overnight in a flask at 80 °C in a vacuum. When the flask cooled, 10 mL of anhydrous DMF was injected under nitrogen. After PEO-PPO-PEO triblock copolymer was dissolved, the DMF solution of PEO-PPO-PEO triblock copolymer was added dropwise during a period of 6 h under nitrogen to 10 mL of anhydrous DMF solution in which CDI (0.83 g, 5.1 mmol) was dissolved, and the mixture was stirred overnight under nitrogen at room temperature. Then, the resulting solution was slowly added dropwise during a period of 3 h into 7.8 g (51 mmol) of tris(2-aminoethyl)amine which was dissolved in 10 mL of anhydrous DMF with stirring at room temperature, followed by stirring the mixture overnight. DMF was removed by vacuum evaporation, and the resulting mixture was dissolved in $CHCl_3$ and washed with H_2O 3 times to remove excess tris(2-aminoethyl)amine. After removing the solvent by evaporation, the product was subjected to column separation using methanol as eluent, to give 2.5 g of PEO-PPO-PEO tetra(amine) as a viscous liquid (yielded, 75%). ^{1}H NMR (300 MHz, DMSO-d_6, δ): 3.35-3.56 (m,82H, and 90H, -CH_2CH_2O- of PEO block and -CH_2CHO- of PPO block), 3.15 (m, 4H, $OCONCH_2$), 2.65 (m, 8H, $CNCH_2$), 2.50 (m, 8H, CH_2N), 1.05 (d, 90H, –CH_3 of PPO block).

1.2 Synthesis of mono-6-azido-6-deoxy-cyclodextrin (β-CD-N_3)

There were two steps of the preparation of β-CD-N_3[2]. In the first step, β-CD (50 g) was dissolved in 300 mL of ultrapure water containing NaOH (5.475) in the 0 °C ice bath. Then, 30

mL of the acetonitrile solution of toluenesulfonyl chloride (mmol/mL) was added slowly. After the reaction for 2 h, the pH of the solution was adjusted to 9.0. After the cryoprecipitation at 4 °C overnight and multiple filtrations and washing, β-CD-Ots was obtained by vacuum drying (yield, 18.5 %). ^{1}H NMR (300 MHz, DMSO, δ): 7.75–7.78 (2H, aromatic protons), 7.45–7.48 (2H, aromatic protons), 5.65–5.89 (14H, OH-2,3), 4.75–4.90 (7H, H-1), 4.20–4.60 (6H, OH-6), 3.45–3.75 (28H, H-3, 5, 6), 3.12–3.42 (14H, H-2,4), 2.40–2.45 (3H, -CH_3 attached to the aromatic ring).

The second step referred to a report [3]. In brief, the dried β-CD-Ots (1 g) was dissolved in 5 mL of DMF, followed by adding 5 mL of NaN_3 aqueous solution of (1.926 mmol/mL). After the reaction at 80 °C for 24 h, the mixture was precipitated and washed with acetone repeatedly. Eventually, β-CD-N_3 was obtained as a white powder (yield, 85%). ^{1}H NMR (300 MHz, DMSO, δ): 5.65–5.89 (14H, OH-2,3), 4.75–4.90 (7H, H-1), 4.20–4.60 (6H, OH-6), 3.45–3.75 (28H, H-3, 5, 6), 3.12–3.42 (14H, H-2,4). ESI-MS (m/z): calculated for C42H69O34N3,1159.0; found, 1182.4 for [M+Na^+].

1.3 Synthesis of β-CD-PAMAM

The prepared β-CD-N_3 and PAMAM-G3 were conjugated through a click reaction to synthesized β-CD-PAMAM, which was set as a control group in the further study. Briefly, β-CD-N_3 (1 g) was dissolved in 20 mL of DMSO, to which 5 mL of PAMAM-G3 aqueous solution (0.186 mmol/mL) was added. After adding copper sulfate pentahydrate (23 mg) for 30 min, 5 mL of sodium ascorbate aqueous solution (92 mg/mL) was added. After the reaction at 70 °C for 72 h, the solution was dialyzed against deionized water for 3 days. The dialysate was freeze-

dried to give β-CD-PAMAM (yield, 64%).

1.4 Synthesis of PEO-PPO-PEO-DNFB

To avoid the assembly between β-CD and PEO-PPO-PEO in the free state, DNBF was used to cap PEO-PPO-PEO. Briefly, aminated PEO-PPO-PEO (0.1 g) was dissolved in 10 mL of DMF, followed by adding DNFB (60 mg). After reacting under the atmosphere of N_2 overnight, the reaction temperature was elevated to 80 °C. After 2 h, the reaction solution was cooled down and concentrated to remove DMF. $CHCl_3$ was used for extraction and then removed by rotary evaporation. Eventually, a column separation was carried out using methanol/dichloromethane as an eluent to give PEO-PPO-PEO-DNFB as a yellow viscous liquid (yield, 79%).

1.5 Synthesis of EPI-PR-PAMAM

To limit the molecule mobility of β-CD on the L64 chain, EPI was used to crosslink β-CD molecules. The obtained EPI-PR was set as a control group in the further study. In brief, PR (1 g) was added in 15 mL of pure water and 20% NaOH solution was added until PR was completely dissolved. Then, the reaction temperature was set as 60 °C, and EPI (1.04 g) was added to the solution slowly. After 24 h, the reaction solution was cooled down and then neutralized with HCl. After the dialysis, EPI-PR was obtained by freeze-drying (yield, 62%).

2. Results and Discussions

2.1 Characterization of β-CD-N_3

First, paratoluensulfonyl chloride was used to sulfonate the hydroxy in position 6 of the

host molecule β-CD, obtaining the monosubstituted β-CD-OTs. Then, NaN_3 was used to replace the active groups of β-CD-OTs to prepare the monosubstituted β-CD-N_3 (**Figure S2**). Chemical structures of β-CD-OTs and β-CD-N_3 were characterized by ^1H NMR with the proton characteristic absorption peaks marked. As shown in Supplementary Figure 2a, in the spectrum of β-CD-OTs, a, b, and c were the proton absorption peaks of tosyl. It was confirmed that only one hydroxyl on a β-CD was substituted by OTs by analyzing integral areas of proton absorption peaks. The ^1H NMR spectra indicated the successful mono-substitution of β-CD. After reacted with NaN_3, the proton absorption peaks of tosyl disappeared. Moreover, confirmed by ESI-MS, the molecule weight of the prepared β-CD-N_3 was consistent with the theoretical molecular weight (Supplementary Figure 2c). As shown in Supplementary Figure 2b, an obvious peak of azide groups appeared at 2110cm^{-1} in the FTIR spectrum[4]. Taken together, mono-subtituted β-CD-N_3 was prepared successfully.

2.2 Characterization of PEO-PPO-PEO tetra(amine)

The obtained polymer PEO-PPO-PEO tetra(amine) was characterized by ^1H NMR (Supplementary Figure 3a) and FTIR (Supplementary Figure 3b). As shown in Supplementary Figure 3a, except for the characteristic peaks of EO and PO, the characteristic peaks of tri(aminoethyl)amine at 3.1, 2.6, and 2.5 ppm were found, confirming the successful modification on PEO-PPO-PEO copolymer by tri(aminoethyl)amine. By analyzing the integral areas of proton absorption peaks of the PO at 1.0 ppm (d) and tri(aminoethyl)amine at 2.5 ppm (g), it could be confirmed that the ends of PEO-PPO-PEO were completely aminated. As presented in Supplementary Figure 3b, the stretching vibrational absorption of primary amine

groups at 3280 cm^{-1}, the characteristic absorption of C=O and N-H in amido linkage at 1640cm^{-1} and 1550cm^{-1}, and the characteristic absorption of C-H at 1365cm^{-1} fully confirmed the successful preparation of PEO-PPO-PEO tetra(amine).

2.3 Characterization of PEO-PPO-PEO-DNFB

To prevent the formation of PPR by β-CD-N_3 and PEO-PPO-PEO, PEO-PPO-PEO tetra(amine) was capped using DNFB and was used as part of the disordered-group. ^{1}H NMR was used to characterize its structure as shown in Supplementary Figure 4. In the spectrum, proton peaks (e, f, g) of DNFB were shown. The area ratio of the proton peak of PEO-PPO-PEO (d) and that of DNFB (g) was calculated as 22:1. Therefore, it was confirmed that DNFB successfully modified the ends of PEO-PPO-PEO.

2.4 Characterization of cationic β-CD (β-CD-PAMAM)

The obtained β-CD-N_3 and PAMAM-G3 were conjugated through a click reaction to prepared β-CD-PAMAM as part of the disordered-group, which was structurally characterized by ^{1}H NMR as shown in Supplementary Figure 5. The peaks of protons in β-CD-N_3 (H^1) and the triazole ring (a) formed by the click reaction suggested the successful preparation of β-CD-PAMAM, and the integral area ratio I_1： Ia was 7:1. Taken together, the above results confirmed that each β-CD-N_3 was conjugated with one PAMAM-G3 through the click reaction.

2.5 Characterization of cationic EPI-PR-PAMAM (Locked-group)

Before the click reaction, EPI was used to couple the host molecules β-CD on the PR chain to

restrict their slide and rotation (Supplementary Figure 6a). The characterization by ^{1}H NMR was shown in Supplementary Figure 6b. It could be found in spectra that the secondary hydroxyl groups between β-CD molecules (β-CD-O-CH_2-C*H*(OH)-CH_2-O-β-CD) showed a characteristic peak at 5.2 ppm[5]. In the FTIR spectra, compared with the spectrum of PR, the spectrum of EPI-PR showed the stretching vibration peaks of methyl and methylene in EPI at 2850 cm^{-1} (Supplementary Figure 6c). Moreover, the stretching vibration peaks of C-O and C-Cl at 1250 cm^{-1} and 800 cm^{-1} were absent, fully confirming that EPI was successfully conjugated to β-CD yielding EPI-PR[6]. A click reaction was carried out for the cationization of EPI-PR using PAMAM-G3, which was characterized by elemental analysis with PR, EPI-PR, and PR-PAMAM as comparisons as shown in Table 1. Compared with PR, the contents of C and H elements increased due to the crosslinking of β-CD molecules by EPI. Moreover, the contents of N in PR-PAMAM and EPI-PR-PAMAM were obviously and higher than that of PR and EPI-PR with a similar increment, revealing the close contents of free PAMAM-G3 and the PAMAM-G3 that were conjugated to the β-CD on EPI-PR.

2.6 Preparation and Characterization of PR

PR was prepared in two steps. First, multiple β-CD-N_3 molecules and aminated PEO-PPO-PEO assembled to form poly(pseudo)rotaxanes (PPR) in H_2O at 20 °C. Second, DNFB was used as the end-capping reagent to prevent β-CD-N_3 fall off and to prepare PR. ITC was used to measure the interaction and molar ratio of the host molecule β-CD-N_3 and the guest molecule PEO-PPO-PEO as shown in Supplementary Figure 7. In the binding isotherm, each peak represented the thermal resulting from a single injection of aqueous β-CD-N_3 into aqueous PEO-PPO-PEO to

form a supramolecular structure. As time prolonged, calories decreased, suggesting that the interaction tended to saturate. The saturated inclusion weight ratio between β-CD-N_3 and PEO-PPO-PEO was determined to be 6.25 by calculating the consumption of β-CD-N_3.

As shown in Supplementary Figure 8, ^{1}H NMR was used for the characterization of PR with β-CD-N_3 as a comparison. In the spectrum of PR, except for the characteristic peaks of EO and PO in PEO-PPO-PEO, characteristic peaks of PR at 5.78, 5.7, and 4.4 ppm, as well as that of the hydroxy (4.8 ppm) and sugar proton (3~4 ppm) in β-CD-N_3, were found, confirming the successful assemble between β-CD-N_3 and the triblock copolymer PEO-PPO-PEO that formed the supramolecular polymer PPR, which was coincident with the above results. Furthermore, the successful end capping reaction was confirmed by the proton characteristic peaks at 8.74, 8.19, and 7.2 ppm of the benzene ring in DNFB. In addition, it was found that there were 13 β-CD-N_3 molecules on each PR chain by analyzing the integral areas of characteristic peaks of protons in PO (1.0 ppm) and β-CD-N_3 (4.8 ppm).

2.7 Preparation and Characterization of PR-PAMAM

An alkyne-azide click reaction was carried out to couple the host molecules β-CD-N_3 on PR and PAMAM-G3, generating the cationic PR-PAMAM. As shown in Supplementary Figure 9, ^{1}H NMR characterized the structure of PR-PAMAM and the proton absorption peaks were marked. In the spectrum of PR-PAMAM, the characteristic peaks of protons in PAMAM-G3 at 2~3 ppm and triazole ring at 7.8 ppm were found, suggesting the successful preparation of PR-PAMAM[7]. Meanwhile, it suggested that 9 β-CD-N_3 molecules on each PR underwent click reactions with PAMAM-G3 by the integral area ratio of the peaks of protons in β-CD-N_3 to

protons in the triazole ring.

2.8 Preparation and Characterization of PR-PAMAM/NONOate

Under the high pressure of NO atmosphere, secondary amines in PR-PAMAM were oxidated for the formation of NONOate to prepare the goal product PR-PAMAM/NONOate. The structural characterization was performed using ^{1}H NMR and FTIR as shown in Supplementary Figure 11. It could be found in spectra that the peak of protons of methylene next to the secondary amine in PAMAM-G3 at 3.09 ppm shifted to 2.73 ppm and 2.54 ppm due to the presence of NONOate. In the FTIR spectrum of PR-PAMAM/NONOate, the vibration absorption peak at 1250 cm^{-1} of νN=N in the NONOate group appeared compared with the spectrum of PR-PAMAM[8].

2.9 Determination of NO

According to the release properties of NONOate carriers, PR-PAMAM/NONOate was immersed in citrate buffer solution (pH 4.0) for the complete release of NO. UV-Vis was used to measure the OD540 using the Griess reagent[9]. As shown in Supplementary Figure 12, the established standard curve $y = 0.0052x - 0.0118$, $R^2 = 0.999$ (y represents OD540, x represents NO^{2-}) was used for the calculation of the NO payload in PR-PAMAM/NONOate, which was confirmed as 0.495 μmol/mg.

3. Supplementary Data

Supplementary Figure 1. Synthetic route of PR-PAMAM/NONOate.

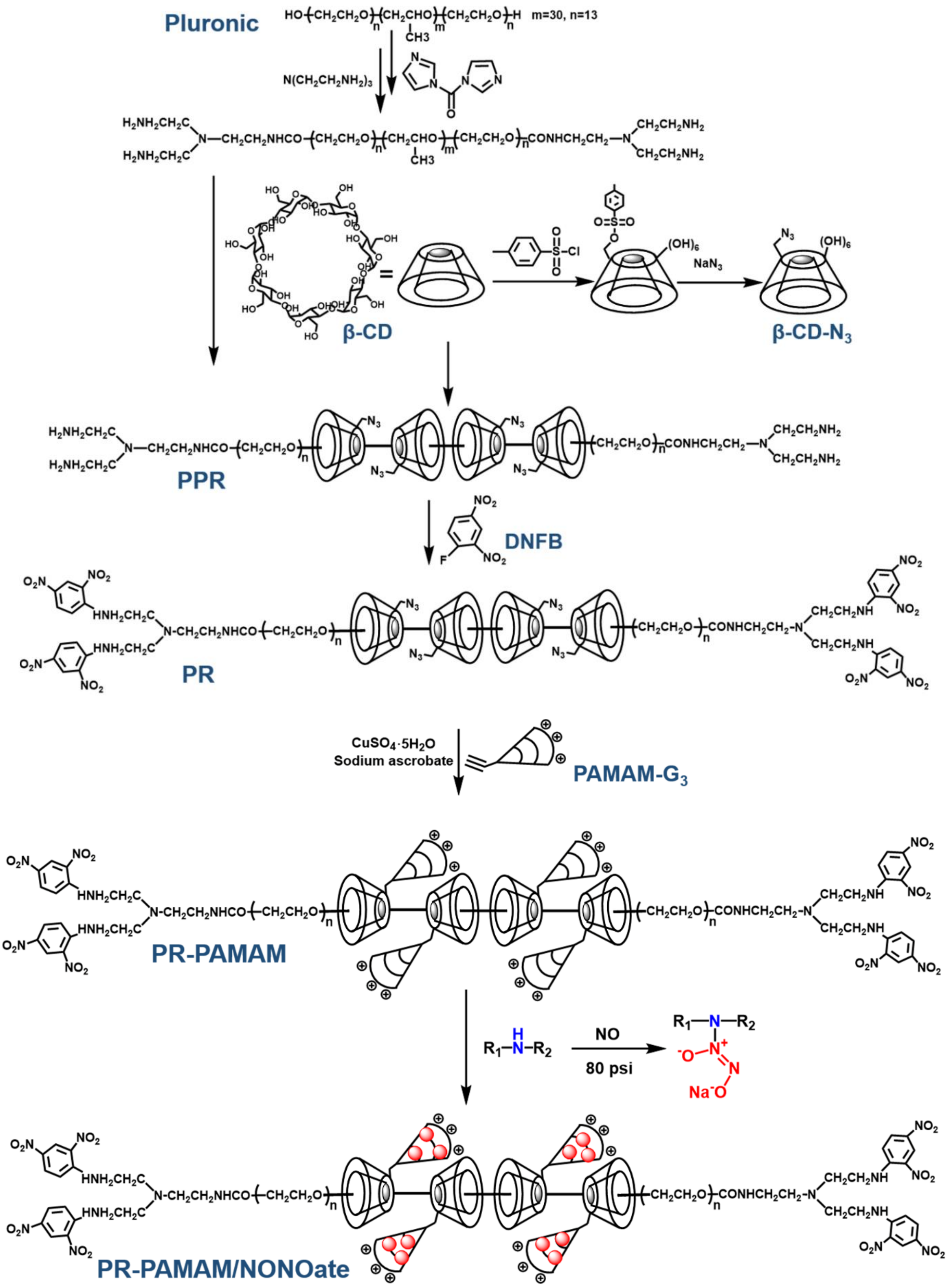

Supplementary Figure 2. (a) ^{1}H NMR spectra of β-CD-OTs and β-CD-N_3 in DMSO at 25 °C. (b) FTIR spectra of β-CD-OTs and β-CD-N_3. (c) The found value of β-CD-N_3 during ESI-MS.

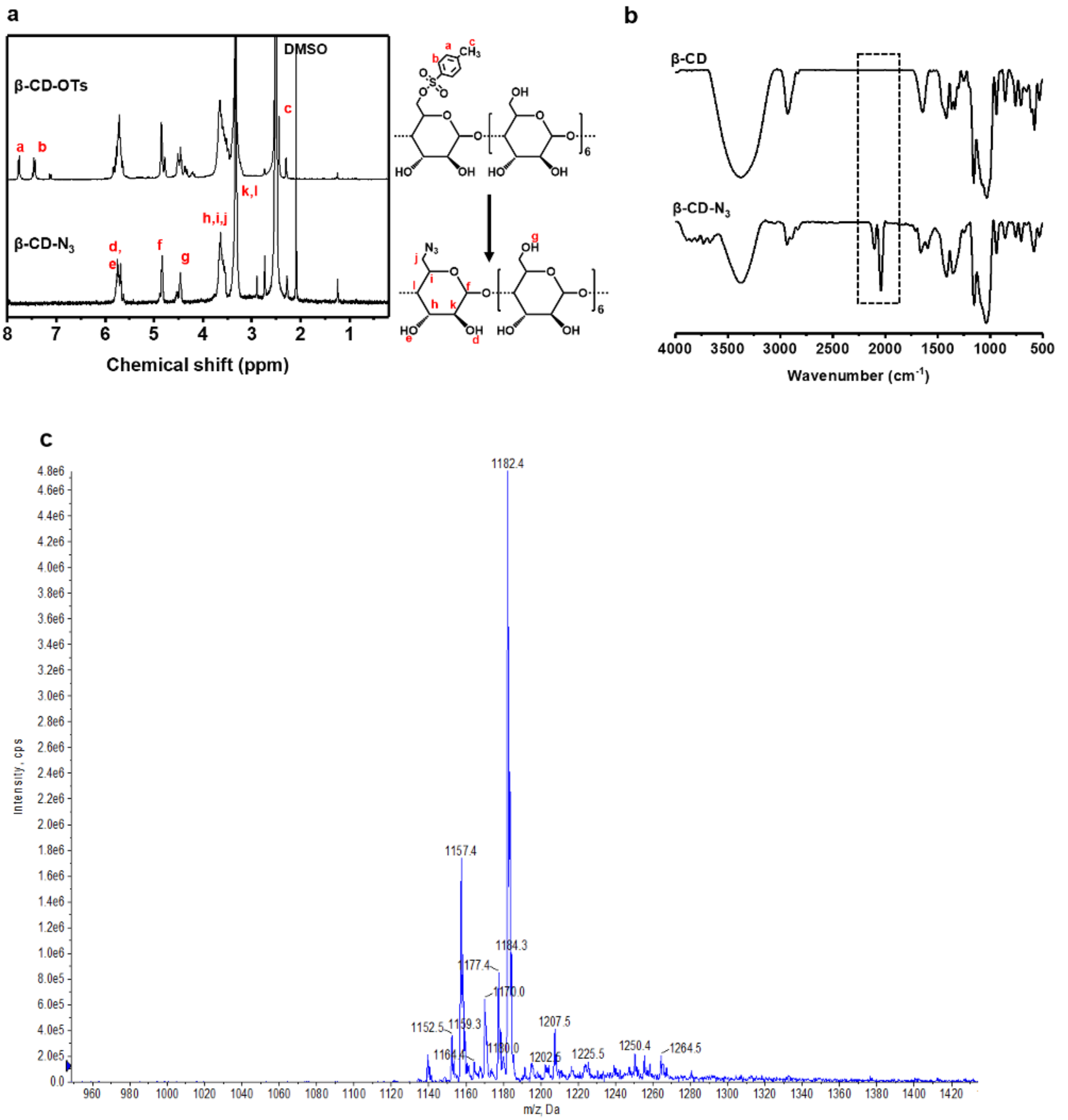

Supplementary Figure 3. (a) ^{1}H NMR spectra of PEO-PPO-PEO and PEO-PPO-PEO tetra(amine) in D_2O at 25 °C. (b) FTIR spectra of PEO-PPO-PEO and PEO-PPO-PEO tetra(amine).

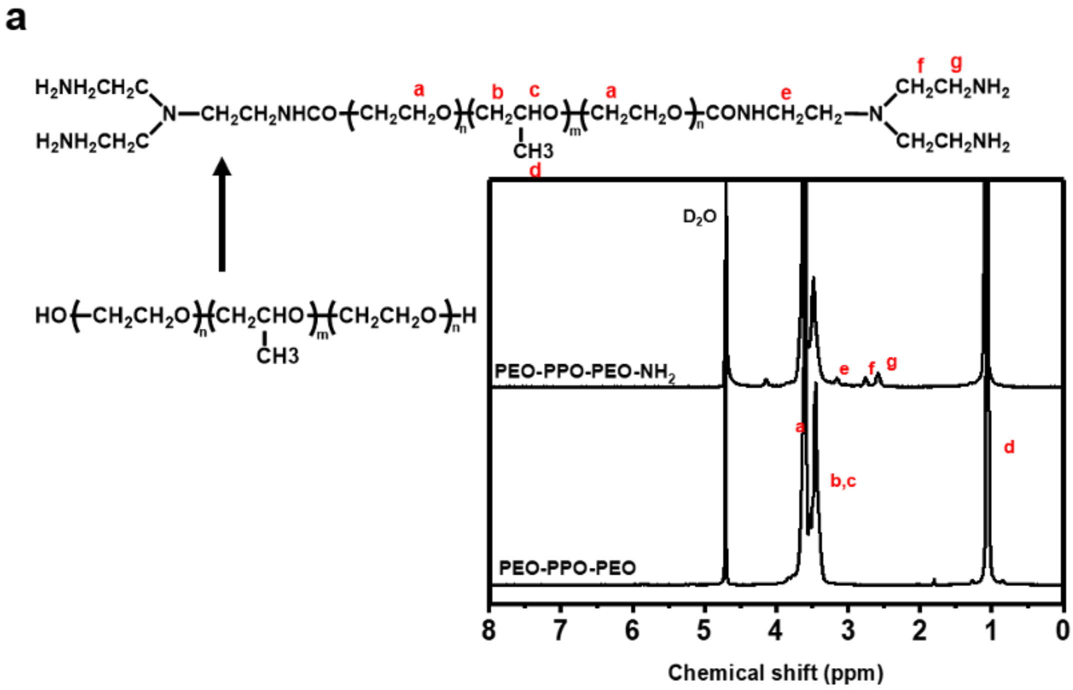

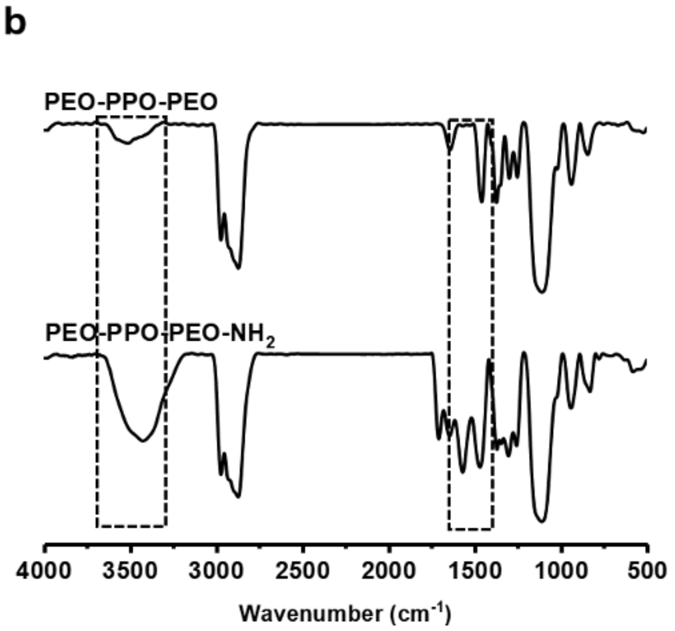

Supplementary Figure 4. ^{1}H NMR spectrum of PEO-PPO-PEO-DNFB.

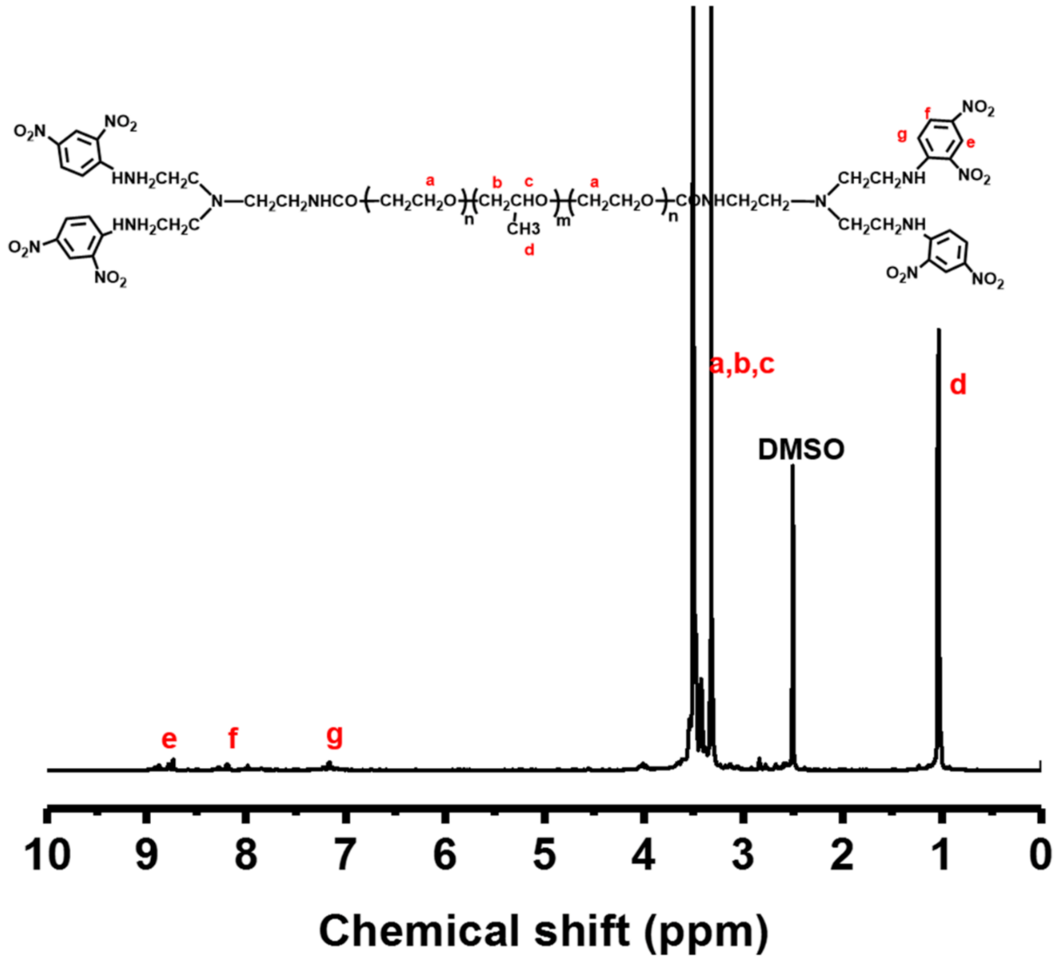

Supplementary Figure 5. ^{1}H NMR spectrum of β-CD-PAMAM.

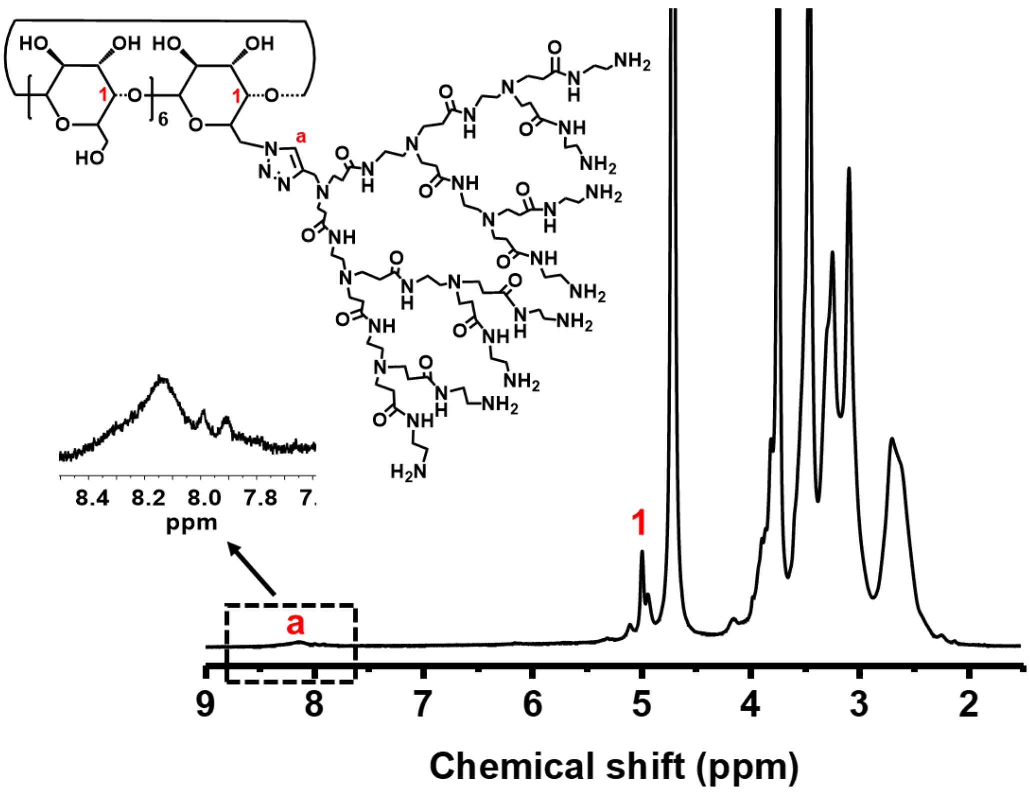

Supplementary Figure 6. (a) The host molecules β-CD on the PR chain was restricted slide and rotation by EPI. (b) ^{1}H NMR spectrum of EPI-PR. (c) FTIR spectra of PR and EPI-PR.

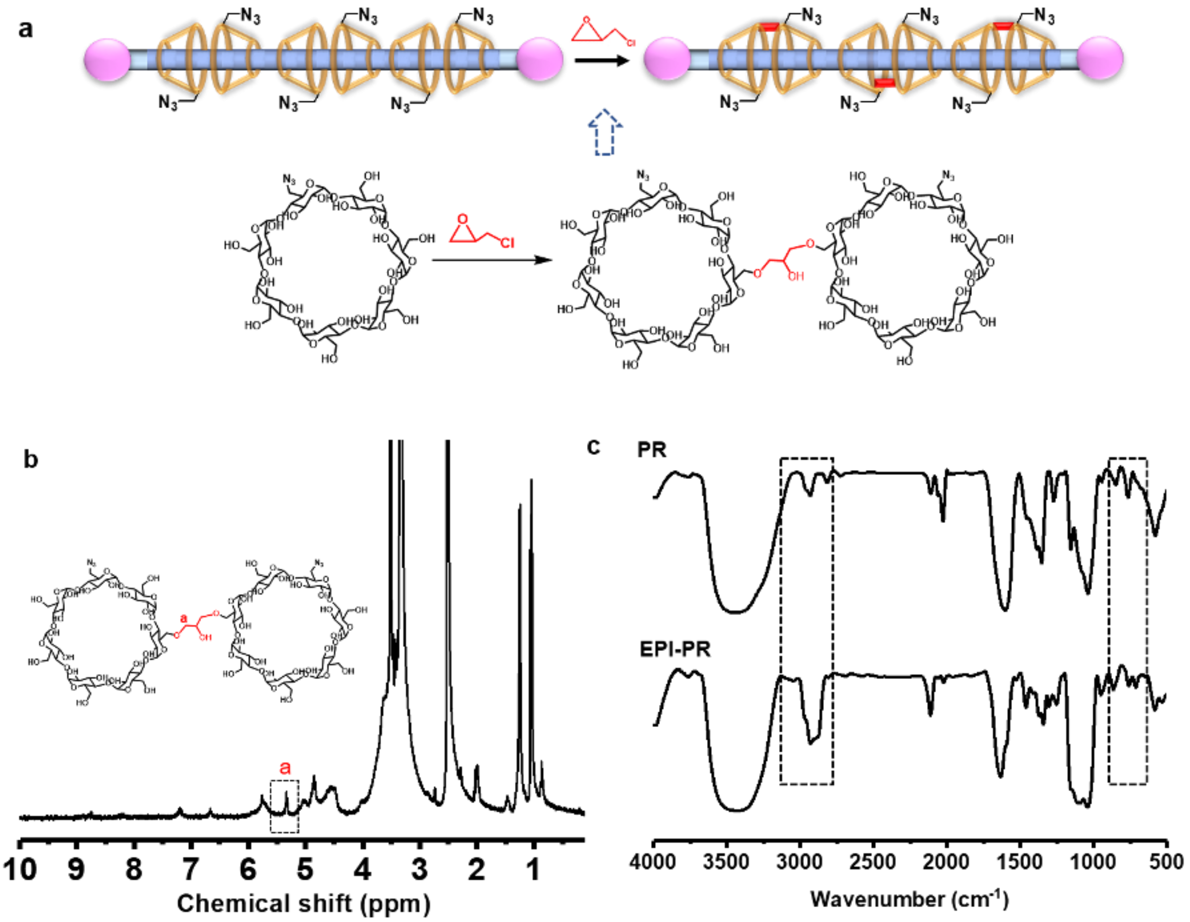

Table 1. Elemental composition (C, N, O) of PR, EPI-PR, PR-PAMAM, and EPI-PR-PAMAM.

	C [%]	N [%]	H [%]
PR	48.58	4.87	4.747
EPI-PR	50.59	4.09	6.603
PR-PAMAM	46.65	13.58	7.444
EPI-PR-PAMAM	48.67	11.35	5.974

Supplementary Figure 7. Typical ITC heat flow trace of the titration of β-CD-N_3 into PEO-PPO-PEO tetra(amine) at 25 °C.

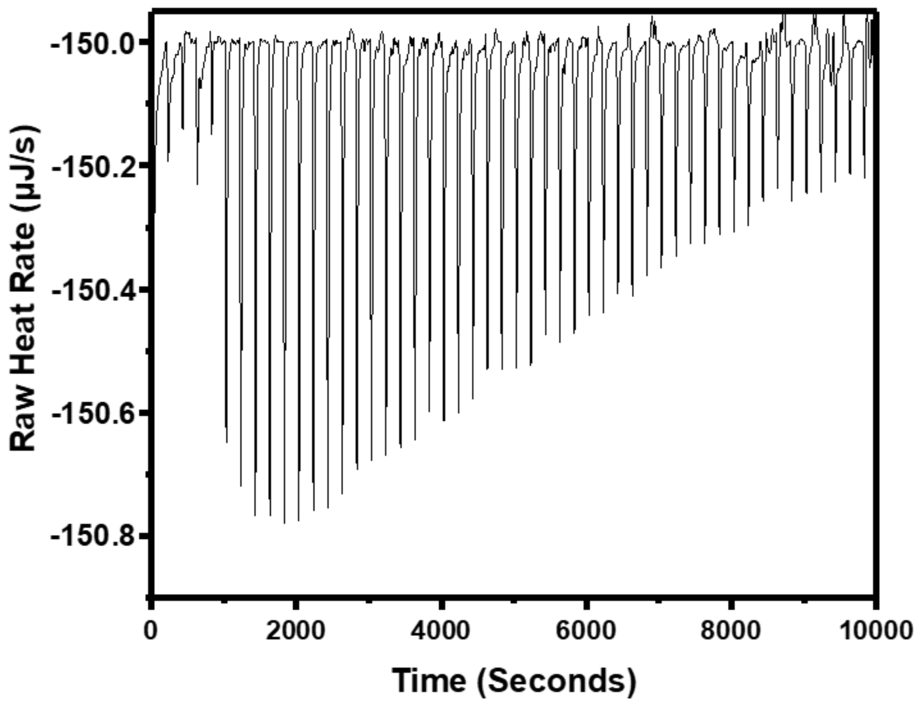

Supplementary Figure 8. ^{1}H NMR spectra of β-CD-N_3 and PR.

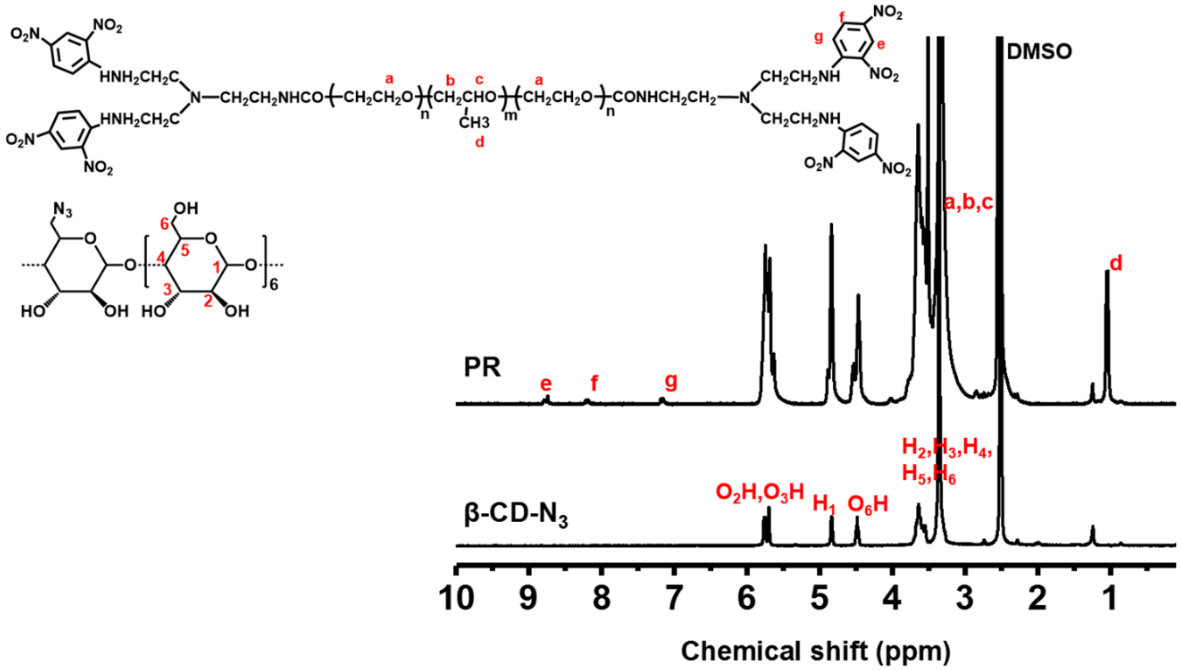

Supplementary Figure 9. ^{1}H NMR spectrum of PR-PAMAM.

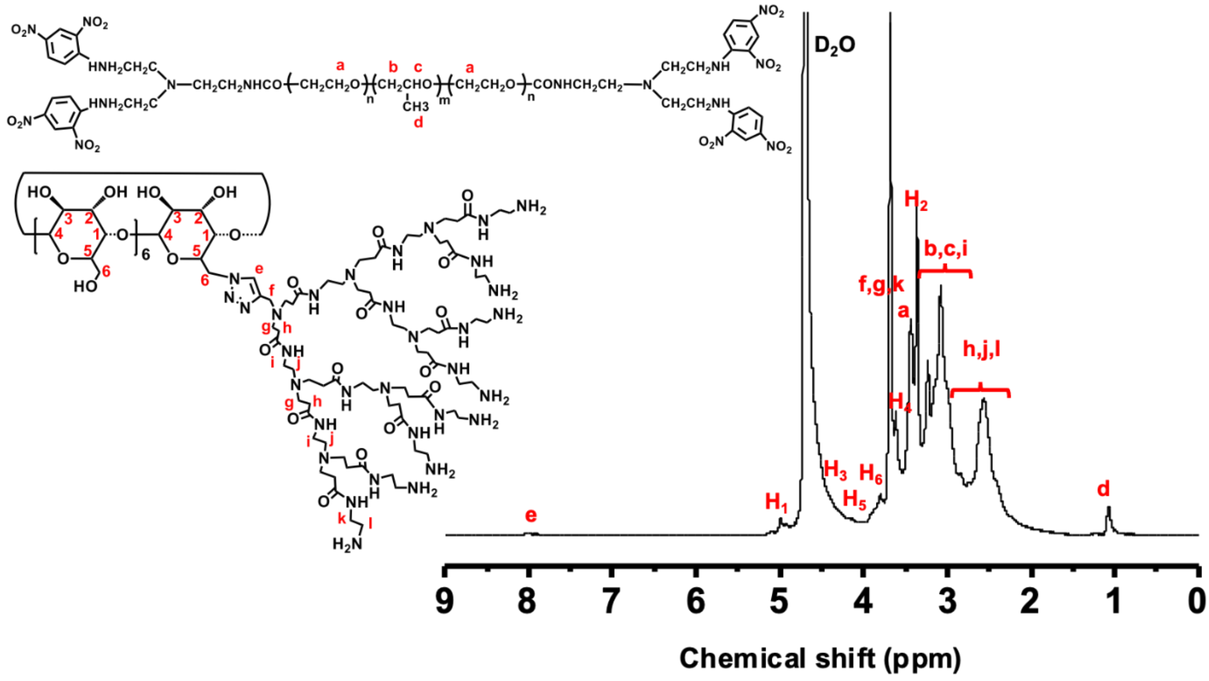

Supplementary Figure 10. Zeta potentials of different nanomaterials (PR-PAMAM, locked-group, and disordered-group).

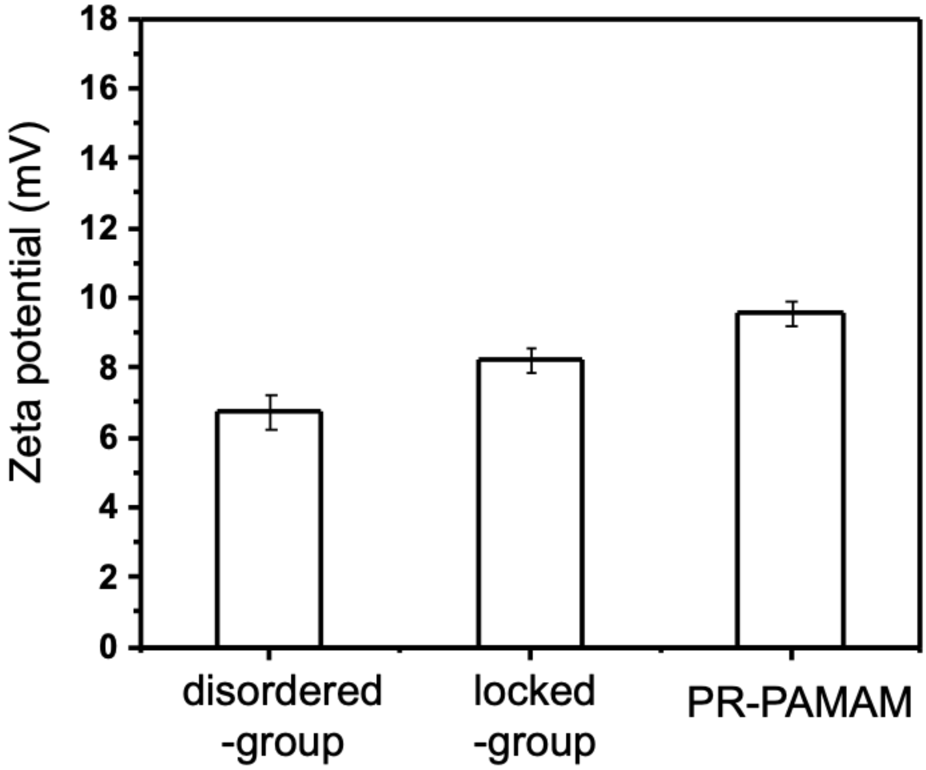

Supplementary Figure 11. (a) ^{1}H NMR spectra for PR-PAMAM and PR-PAMAM/NONOate. (b) FTIR spectra of PR-PAMAM and PR-PAMAM /NONOate.

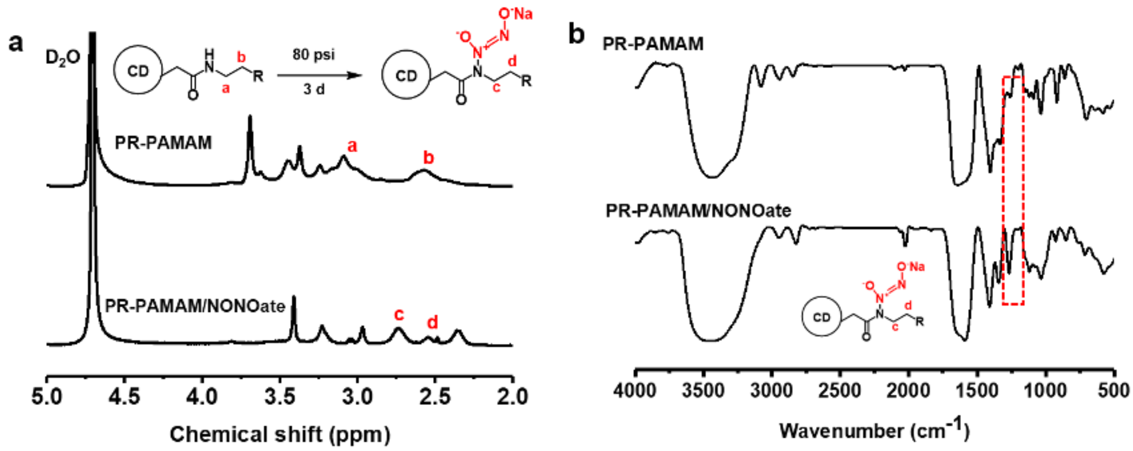

Supplementary Figure 12. Calibration curve (data presented as mean ± standard deviation, n = 3).

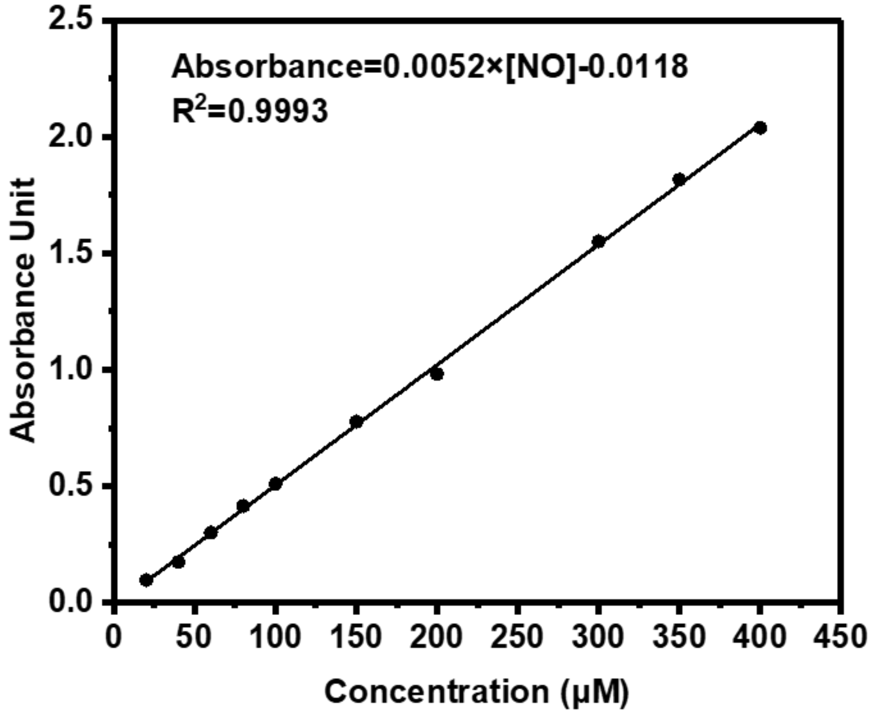

Supplementary Figure 13. Cumulative release of NO from PR-PAMAM/NONOate in PBS (pH 7.4) at 37℃.

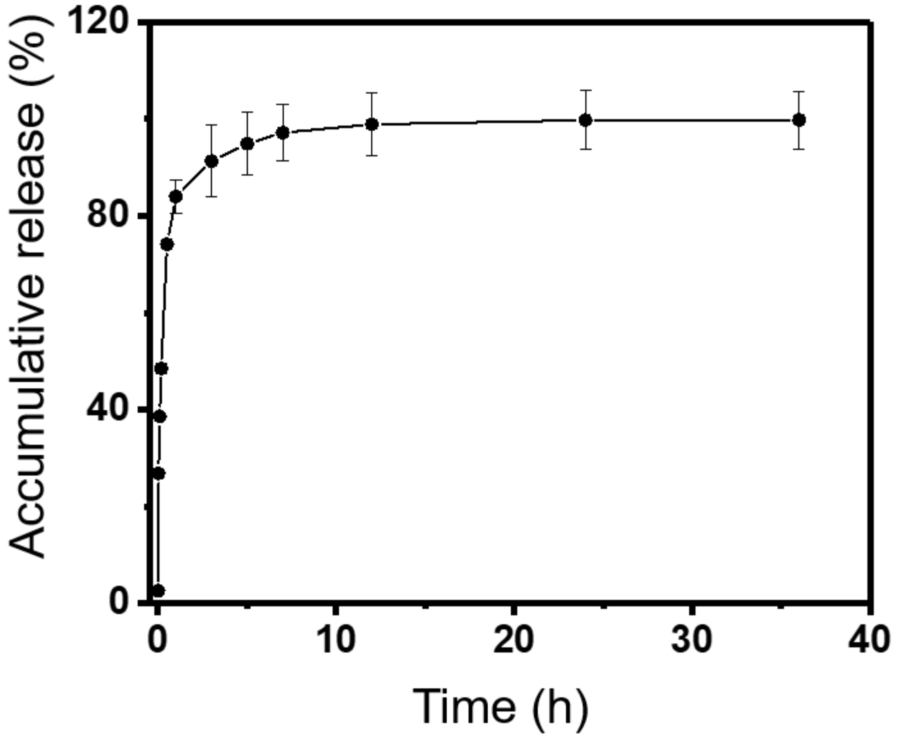

Supplementary Figure 14. (a) Fluorescence intensity at 650/670 nm of Cy5.0 labeled materials (50 μg/mL). (b) Fluorescence images of MRSA incubated with different formulations at 0, 0.5, 1, and 2 h.

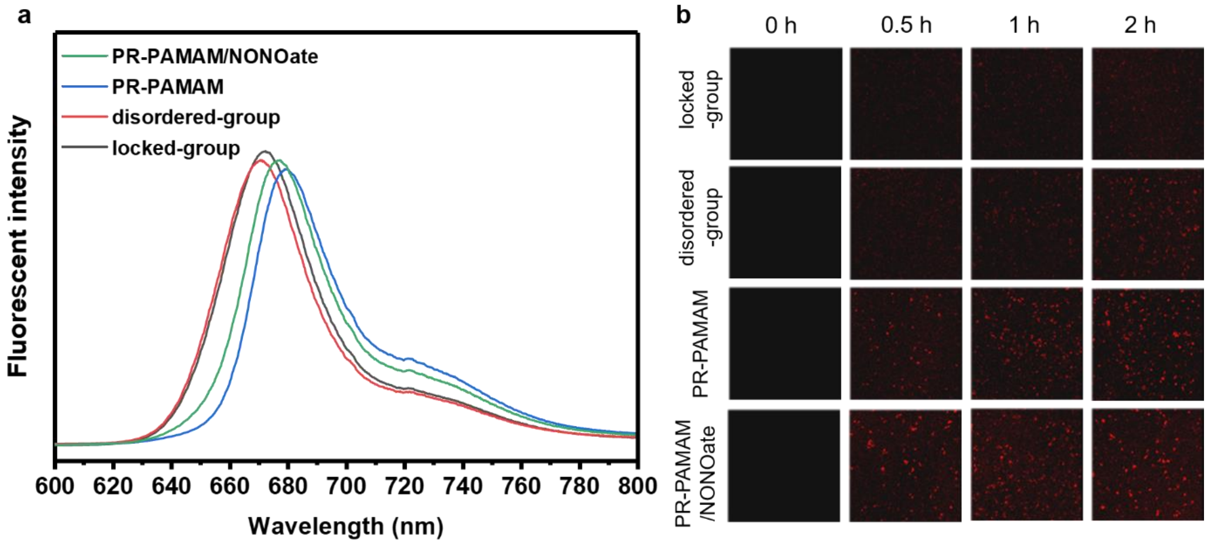

Supplementary Figure 15. (a) Digital images of the bacterial colonies in agar plate after different treatments on different days. (b) Histological images with H&E, Masson, and Giemsa staining of wound tissue collected after 3 days of different treatments. Scale bars represent 50 μm.

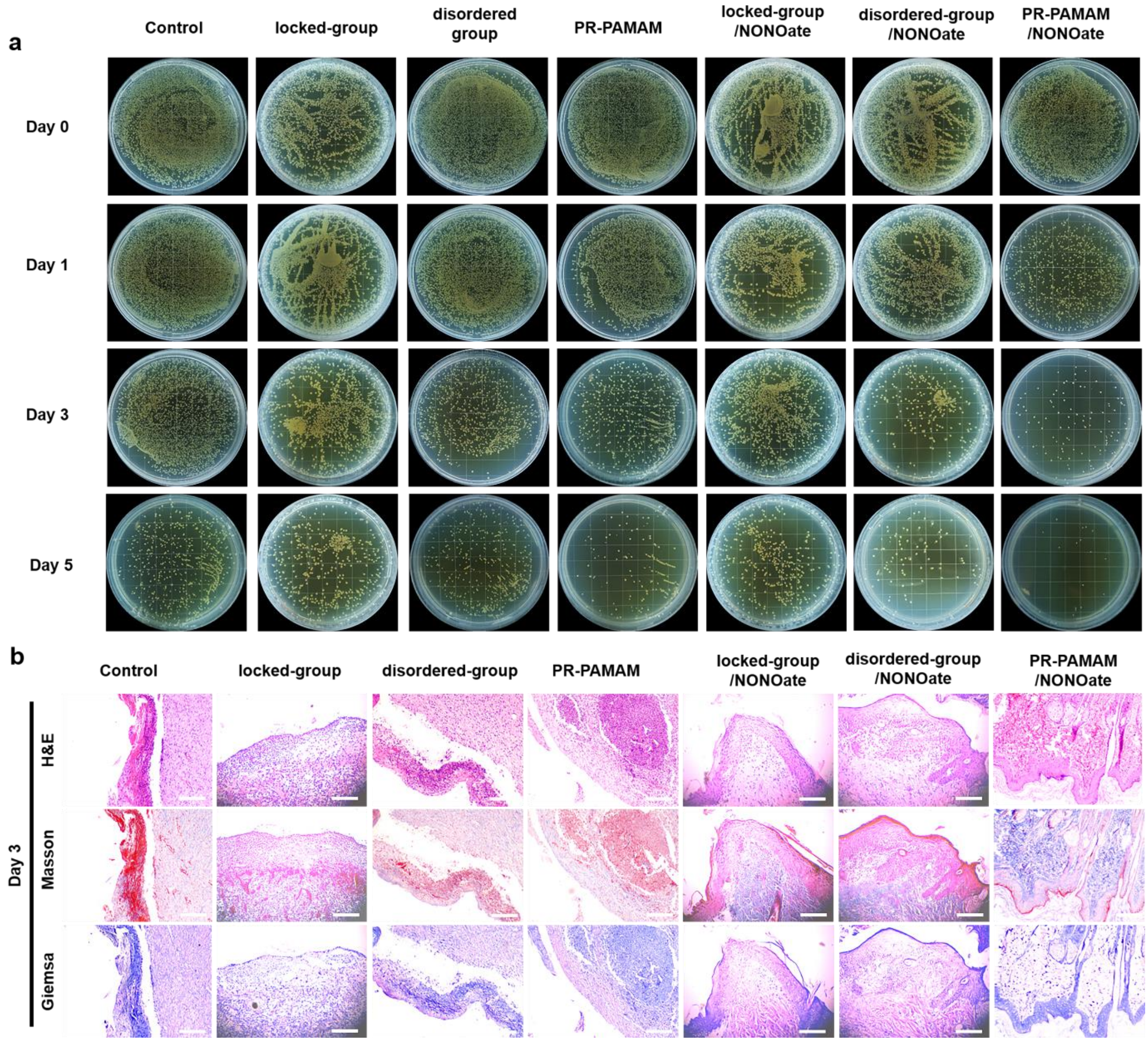

Supplementary References

[1] J. Li, C. Yang, H. Li, X. Wang, S. H. Goh, J. L. Ding, D. Y. Wang, K. W. Leong, *Adv. Mater.* **2006**, 18, 2969.

[2] H. Jin, L. Yang, M. J. R. Ahonen, M. H. Schoenfisch, *J. Am. Chem. Soc.* **2018**, 140, 14178.

[3] W. Tang, S.-C. Ng, *Nat. Protoc.* **2008**, 3, 691.

[4] T. Liu, G. Li, X. Wu, S. Chen, S. Zhang, H. Han, H. Zhang, X. Luo, X. Cai, D. Ma, *Drug delivery* **2021**, 28, 306.

[5] A. Harada, M. Furue, S.-i. Nozakura, *Polym. J.* **1981**, 13, 777.

[6] P. K. Papaioannou, C. S. Karagianni, G. Kakali, V. G. Charalampopoulos, *J. Phys. Chem. Solids* **2018**, 114, 246.

[7] Q. Lin, Y. Yang, Q. Hu, Z. Guo, T. Liu, J. Xu, J. Wu, T. B. Kirk, D. Ma, W. Xue, *Acta Biomater.* **2017**, 49, 456.

[8] S. Liu, X. Cai, W. Xue, D. Ma, W. Zhang, *Carbohydr. Polym.* **2020**, 234, 115928.

[9] G. Li, S. Yu, W. Xue, D. Ma, W. Zhang, *Chem. Eng. J.* **2018**, 347, 923.
